# Filamin A Phosphorylation at Serine 2152 by the Serine/Threonine Kinase Ndr2 Controls TCR-Induced LFA-1 Activation in T Cells

**DOI:** 10.3389/fimmu.2018.02852

**Published:** 2018-12-04

**Authors:** Natalie Waldt, Anke Seifert, Yunus Emre Demiray, Eric Devroe, Benjamin E. Turk, Peter Reichardt, Charlie Mix, Annegret Reinhold, Christian Freund, Andreas J. Müller, Burkhart Schraven, Oliver Stork, Stefanie Kliche

**Affiliations:** ^1^Institute of Molecular and Clinical Immunology, Health Campus Immunology, Infectiology and Inflammation (GC-I^3^), Otto-von-Guericke-University, Magdeburg, Germany; ^2^Institute of Biology, Department of Genetics and Molecular Neurobiology, Otto-von-Guericke University, Magdeburg, Germany; ^3^MD Anderson Cancer Center, University of Texas, Houston, TX, United States; ^4^Department of Pharmacology, Yale School of Medicine, New Haven, CT, United States; ^5^Protein Biochemistry Group, Institut für Chemie und Biochemie, Freie Universität Berlin, Berlin, Germany; ^6^Intravital Microscopy of Infection and Immunity, Helmholtz Centre for Infection Research, Braunschweig, Germany; ^7^Department of Immune Control Helmholtz Center for Infection Research, Braunschweig, Germany; ^8^Center for Behavioral Brain Sciences, Magdeburg, Germany

**Keywords:** LFA-1, TCR, Ndr2, Filamin A, Talin, Kindlin-3, inside-out signaling, T cells

## Abstract

The integrin LFA-1 (CD11a/CD18) plays a critical role in the interaction of T cells with antigen presenting cells (APCs) to promote lymphocyte differentiation and proliferation. This integrin can be present either in a closed or in an open active conformation and its activation upon T-cell receptor (TCR) stimulation is a critical step to allow interaction with APCs. In this study we demonstrate that the serine/threonine kinase Ndr2 is critically involved in the initiation of TCR-mediated LFA-1 activation (open conformation) in T cells. Ndr2 itself becomes activated upon TCR stimulation and phosphorylates the intracellular integrin binding partner Filamin A (FLNa) at serine 2152. This phosphorylation promotes the dissociation of FLNa from LFA-1, allowing for a subsequent association of Talin and Kindlin-3 which both stabilize the open conformation of LFA-1. Our data suggest that Ndr2 activation is a crucial step to initiate TCR-mediated LFA-1 activation in T cells.

## Introduction

T lymphocytes require dynamic regulation of adhesive contacts for their interaction with other cell types. An important mediator of these functions is the integrin lymphocyte function-associated antigen-1 (LFA-1; CD11a/CD18), which is required for arrest of rolling lymphocytes on the endothelium, extravasation into lymph nodes or inflamed tissue and interactions with antigen presenting cells (APCs) (1).

On non-activated T cells, LFA-1 is maintained in a closed conformation with low affinity for its ligand, the Immunoglobulin-like Cell Adhesion Molecule-1 and 2 (ICAM-1/2). Stimulation of the T-cell receptor (TCR) by Ag/MHC-complexes induces a conformational opening of LFA-1 that increases its affinity for ICAM-1 and facilitates avidity modulation (clustering) of LFA-1, a process termed “inside-out signaling” (1–3). Subsequently, extracellular binding of the ICAM-1 ligands to LFA-1 provides a co-stimulatory signal to the T cells to drive their activation, differentiation and proliferation (“outside-in signaling”) (1, 4).

Three intracellular binding partners of LFA-1, i.e., Filamin A (FLNa), Kindlin-3 and Talin have been reported to regulate inside-out-signaling of LFA-1 in T cells (1). While FLNa-deficiency in T cells increases T-cell adhesion and LFA-1 activation (5, 6), loss of Talin or Kindlin-3 in lymphocytes abolishes T-cell adhesion to ICAM-1 and affinity regulation of LFA-1 (7–11). Talin, Kindlin-3 and FLNa bind to overlapping sites within the cytoplasmic tail of the LFA-1 β2-chain (CD18) (12, 13). Moreover, Talin competes with FLNa for binding to the cytoplasmic tail of CD18 *in vitro* (14). The current model of LFA-1 activation therefore proposes that in non-activated T cells FLNa is bound to LFA-1 keeping the integrin in an inactive (closed) conformation. Upon TCR-stimulation, FLNa dissociates from CD18, Talin and Kindlin-3 are recruited to the plasma membrane and interact with LFA-1 to promote the activated (open) conformation. Thus, the dissociation of FLNa from LFA-1 appears to be a critical step in this activation process. However, the molecular mechanisms and the intracellular signals that control the release of FLNa from CD18 are not sufficiently understood.

The small GTPase Rap1 is key regulator of integrin activation (15). Activated Rap1 binds to the Rap1 effector proteins regulator for cell adhesion and polarization enriched in lymphoid tissue (RAPL) and Rap1–GTP interacting adapter molecule (RIAM) (16–18). Another critical component for TCR-regulated inside-out signals is a complex consisting of the two cytosolic adapter proteins adhesion and degranulation promoting adapter protein (ADAP) and src kinase-associated phosphoprotein of 55 kDa (SKAP55) (19, 20). Loss of these proteins attenuates TCR-mediated adhesion and interaction with APCs (21–23). In this complex SKAP55 constitutively interacts with RAPL or RIAM (24, 25). The loss of SKAP55 or disruption of these interactions abrogates membrane targeting of RAPL, RIAM, and Talin and also their interaction with LFA-1 (24–28). Moreover, SKAP55 also participates in outside-signaling events regulating LFA-1-mediated de-adhesion (29).

In addition RAPL interacts with the Ste20-like kinases Mst1, a core component of the so-called Hippo pathway (30). Loss of Mst1 attenuates TCR-mediated affinity regulation of LFA-1, T-cell adhesion and interaction with APCs (10, 31–33). Mst signals are mediated, in part, by the Nuclear Dbf2-related kinases (Ndr) 1 and Ndr2 (34, 35), which are widely expressed in mammalian tissues including hematopoietic organs cells (36, 37). Previous studies have demonstrated that Ndr1/2 control centrosome duplication and alignment, cell-cycle exit, apoptosis, cell polarity and proliferation (34, 35). Importantly, aged Ndr1-deficient mice spontaneously develop T-cell lymphomas (38), whereas T cells from young Ndr1/2-defcient mice are defective in thymocyte egress and T-cell homing (36). Kondo et al. recently showed that Ndr1 regulates TCR-mediated LFA-1 affinity by binding to Kindlin-3 and recruitment to LFA-1 (10). In line with these observations, we previously showed that Ndr2 controls integrin-activation and integrin-dependent differentiation in neuronal cells (39, 40). This led us to hypothesize that Ndr2 might play a critical role in TCR-mediated LFA-1 activation.

Therefore, we investigated the activation of Ndr2 upon TCR-stimulation and the critical involvement of its kinase activity in TCR-mediated signaling processes involved in LFA-1 activation. We identified FLNa as a substrate of Ndr2 *in vitro* and demonstrated that Ndr2 phosphorylates FLNa at serine 2152 (S2152) upon TCR-triggering *in vivo*. We also show that Ndr2-dependent phosphorylation of FLNa at S2152 releases the binding of this molecule from the inactive conformation of LFA-1, thus allowing the TCR-mediated association of Talin and Kindlin-3 to the cytoplasmatic domain of CD18. Our data suggest that phosphorylation of FLNa at S2152 by Ndr2 is a critical step in TCR-mediated LFA-1 activation.

## Materials and Methods

### Mice, Isolation of Lymphocytes, Cell Culture and Transfection

C57BL/6, BALB/c, D011.10, and Ndr2^−/−^ (40) mice were bred and maintained under specific pathogen-free conditions at the Otto-von-Guericke University, Magdeburg, Germany, according to the guidelines of the State of Sachsen-Anhalt, Germany. Animal maintenance and tissue collection were done according to the guidelines of the State of Saxony-Anhalt, Germany and approved by the Landesverwaltungsamt Sachsen-Anhalt (IMKI-TWZ-02). Primary human T cells, splenic pan or CD4^+^ T cells as well as B cells from mice were purified using the appropriated T- or B-cell isolation kits and AutoMacs magnetic separation system (Miltenyi Biotec). Approval for the isolation of human T cells from healthy donors was obtained from the Ethics Committee of the Medical Faculty at the Otto-von-Guericke University (79/13), Magdeburg, Germany. Informed consent was obtained in accordance with the Declaration of Helsinki. Jurkat T cells (JE6.1 ATCC) and B cells (Raji; ATCC) were maintained in RPMI 1640 medium (Biochrom AG) supplemented with 10% fetal bovine serum (FBS, PAN) and stable L-glutamine at 37°C with 5% CO_2_. Jurkat T cells (2x10^7^ cells) were transfected with plasmids by electroporation, as previously described (41). Transfection with the pCMS4 plasmids into Jurkat T cells consistently yielded in an average of app. 80% GFP-expressing cell population after 48 h. HEK 293T cells were cultured in DMEM medium containing 10% FBS and penicillin/streptomycin (100 U/ml/100 μg/ml; Biochrom AG). HEK 293T cells were transiently transfected using the standard calcium phosphate precipitation method. When indicated, transfected cells were treated with 1 μM okadaic acid (OA; Calbiochem Merck) for 1 h prior to harvesting.

### cDNA Constructs and Generation of Plasmids

cDNA constructs for human Ndr1, Ndr2 and its K119A mutant cloned into the pECMV vector were described and generously provided by Pamela A. Silver [Wyss Institute at Harvard University, USA (42)]. Full-length Mob2 inserted into the pEGFP-C1 vector has been previously described (42). The dsRed-tagged cDNA constructs of wild type Filamin A (FLNa) and its mutants (S2152A and S2152E) were kindly provided by Miguel A. Del Pozo [Department of Vascular Biology, Centro Nacional de Investigaciones Cardiovasculares (CNIC), Spain; (43)]. The dsRed-C1 vector was obtained from Clontech. The pGEX 4-T-1 vector was purchased from GE Healthcare. Repeats 19–24 of human FLNa cloned in the vector pGEX 4-T-1 was given by M. Humphries [Wellcome Trust Centre for Cell-Matrix Research, University of Manchester, UK (44)]. The pGEX CD18 cytoplasmatic domain has been described (45). To target FLNa with shRNA the 21-nucleotide sequence CCCACCCACTTCACAGTAAAT was used to target by shRNA (FLNa shRNA for/FLNa shRNA rev) and cloned into the pCMS4 vector (a gift from D. Billadeau; Department of Biochemistry and Molecular Biology, Division of Oncology Research, Mayo Clinic, Rochester, MN, USA). The cDNA sequences of Ndr1 and Ndr2 were amplified to introduce the two restriction sites BglII and XbaI needed for cloning into the pEF-BOS-FLAG vector or pEGFP-C1 vector (Ndr2 BglII for/Ndr2 SalI/XbaI rev and Ndr1 BglII for/Ndr1 SalI/XbaI rev). To target Ndr2 by shRNA the 19-nucleotide sequence GAGGAAACACAGTTCTACA (Ndr2 shRNA for/Ndr2 shRNA rev) was used and cloned into the pCMS4 vector. For the construction of the “suppression/re-expression” plasmids full-length Ndr2 cDNA was amplified and two restriction sites for Mlu1 and Not1 were introduced to facilitate cloning into the pCMS4 vector (Ndr2 MluI for/Ndr2 Not I rev). The QuikChange site-directed mutagenesis kit (Agilent Technologies) was used to mutate the shRNA target sequence to GAaGAgACgCAaTTtTAtA (lowercase letters indicates the changed nucleotide that do not affect the amino acid sequence) with the primer pair Ndr2 sh-res for/Ndr2 sh-res rev. The primer pair Ndr2 K119A for/Ndr2 K119A rev was used to generate a kinase-dead mutant of Ndr2 where lysine 119 was mutated to alanine [K119A (42)]. All amplified PCR products were cloned into the pJET1.2 vector (Thermo Science) and sequenced prior to sub-cloning in the designated vectors or after mutagenesis. Please refer to Table S1 for sequences of primers used for cloning, PCR and mutagenesis.

### Antibodies

The anti-human CD3 monoclonal antibodies (mAb) (clone OKT3; eBioscience or clone C305) and the anti-mouse CD3 mAb (non or biotinylated; clone 145-2C11; eBioscience) were used for T-cell stimulation. The anti-human CD18 mAb (clone MEM48; antibody-online.de), anti-human CD3 (clone OKT3) or mAb24 (provided by N. Hogg; Cancer Research U.K. London Research Institute, London, U.K.) were used for flow cytometric analysis of Jurkat T cells. The following fluorescently labeled Abs were used for purified splenic CD4^+^ T cells: CD4 (clone RM4-5), CD3 (clone 145-2C11), CD18 (clone M18/2) CD11a (clone M17/4) and CD69 (clone H1.2F3) including the appropriated isotype controls. The anti-CD11a (clone M17/4) mAb was used for blocking studies. These antibodies were purchased from BioLegend. For Western blotting and immunoprecipitation the following antibodies (Ab) were used: anti-FLAG M2 mAb, anti-β-actin mAb, anti-Talin mAb (clone 8D4) (all from Sigma), anti-Kindlin-3 mAb (Abcam), anti-FLNa mAb, anti-GFP mAb (both from Santa Cruz), anti-FLNa rabbit (Abcam), anti-pFLNa S2152 rabbit Ab, anti-pERK1/2 Thr202/Y204 rabbit Ab, anti-pMst1/2 (Thr183)/(Thr180) rabbit Ab (all from Cell signaling), anti-CD11a mAb (clone 38; Calbiochem Merck), anti-Mst1 mAb (from BD Bioscience) and anti-dsRed mAb (ChromoTek GmbH). Because human Ndr1 and Ndr2 share ~87% identity at the amino acid level (42), the anti-Ndr2 mAb (clone 4D8; Origene), the anti-Ndr1 goat Ab (MyBioSource), and the Ndr2 rabbit Ab (46) were tested for their specificity to detect either Ndr1 or Ndr2 by Western blotting (Figure S1A). In addition, the Ndr2 rabbit Ab was used for immunoprecipitation studies and the specificity of this antibody to recognize only Ndr2 but not Ndr1 was addressed as depicted in Figure S1B.

### Protein Purification

GST, GST-tagged Igl repeats 19–24 of human FLNa and GST-tagged cytoplasmic domain of CD18 (GST-CD18_cyt_) were expressed in BL21 (DE3) cells and purified using glutathione-sepharose (Novagen or GE Healthcare) according to the manufacturer's instructions. Purity of these proteins was assessed by SDS-PAGE followed by Commassie staining (Figure S2).

### Flow Cytometry, mAb24 Binding Assay and T Cell Activation *in vitro*

To analyze the cell surface expression of CD18 and CD3, Jurkat T cells were stained with the indicated antibodies in combination with FITC/APC-conjugated goat anti-mouse IgG (Dianova). mAb24 binding was assessed as previously reported (47). Briefly, cells were incubated for various time points on plate-bound anti-IgG2a antibody or with plate-bound anti-CD3 antibody in the presence of plate-loaded human Fc-tagged ICAM-1 (10 μg/ml; R&D system) and mAb24 antibody (10 μg/ml). Bound mAb24 antibody was detected using FITC/APC-conjugated anti-mouse IgG1 (Dianova) and analyzed by flow cytometry. Epitope expression of mAb24 was normalized against LFA-1 expression detected by MEM48. To analyze the cell surface expression of purified CD4^+^ T cells for LFA-1 (CD18 or CD11a) or the TCR, cells were stained with the indicated Abs. For T-cell activation *in vitro* isolated splenic CD4^+^ T cells were stimulated with plate-bound anti-CD3 mAbs (0.1 μg/ml clone 14-2C11) in the absence or presence of plate-bound mouse ICAM-1 Fc chimera (5 μg/ml) with or without blocking LFA-1 mAbs (15 μg/ml clone M17/4) for 12 h. Untreated (0 h) or stimulated cells (12 h) were stained with Abs for the activation marker CD69. Ab-labeled T cells were analyzed using a FACSCalibur flow cytometer and CellQuestPro software (BD Biosciences).

### Adhesion and Conjugation Assay

Adhesion assays were performed using a 96-well plate pre-coated with 0.5 μg of the integrin ligand recombinant human or mouse ICAM-1/CD54 Fc chimera/well (R&D Systems). Purified splenic CD4^+^ T cells or transfected Jurkat T cells were left untreated or stimulated with anti-CD3 mAb [145-2C11 (5 μg/ml) or OKT3 (5 μg/ml)] for 30 min at 37°C prior to the adhesion assay. Cells were then allowed to adhere for 30 min at 37°C, unbound cells were carefully washed off with Hanks buffered saline (HBSS, Biochrom AG). Bound cells were counted and calculated as percentage of input (2 × 10^5^ Jurkat T cells or 1 × 10^6^ mouse T cells) in duplicates or triplicates (47, 48). Conjugate formation was performed as previously described (24, 49). Briefly, untreated or staphylococcal enterotoxin E-pulsed and DDAO-SE-labeled Raji B cells were incubated with an equal number of Jurkat T cells for 30 min at 37°C. Nonspecific aggregates were disrupted; cells were fixed with 1% PFA, and then analyzed by flow cytometry. The percentage of conjugates was defined as the number of double-positive events in the upper right quadrant. For confocal microscopy purified splenic B cells from BALB/c mice were loaded with 100 μg/mL ovalbumin 323-339 peptide, washed and mixed at a ratio 1:1 ratio with freshly isolated splenic CD4^+^ T cells from DO11.10 mice. Cells were incubated for 30 min at 37°C on poly-L-Lysine-coated slides (Marienfeld AG) and fixed with 3.5% paraformaldehyde (PFA) in PBS for 10 min. Cells were permeabilized with 0.1% Triton X-100 in PBS, blocked with 5% horse serum in PBS, and incubated with Ndr2 rabbit Abs and Cy3-labeled CD3 mAb clone 145-2C11) or Ndr2 rabbit Abs in combination with TRITC-conjugated phalloidin (Sigma Aldrich). Bound Ndr2 antibodies were detected with FITC-conjugated goat anti-rabbit IgG (Dianova). Coverslips were mounted in Mowiol 488 and imaged with a LEICA TCS SP2 laser-scanning confocal system (Leica Microsystems) with a Leica IRE2 stand equipped with a 63× 1.40 HCX APO CS objective (Leica), an argon laser (488 nm), and a HeNe laser (543 nm). To avoid spectral overlap, the samples were sequentially scanned with a beam splitter HFT 488/543/633. Green and red fluorescence emission were read out at 500 to 551 nm and 571 to 626 nm, respectively. Figure constructions of images were performed in COREL Photopaint.

### Western Blotting, Immunoprecipitation and GST Fusion Protein Pull-Down Assay

HEK 293T cells, Jurkat T cells or purified splenic CD4^+^ T cells were left untreated or stimulated for various time points with OKT3, C305 or biotinylated 14-2C11 mAbs and lysed as previously described (24, 41, 49, 50). Equivalent amounts of protein [determined by Bradford assay (Carl Roth)] were used for precipitation studies (200–500 μg of total protein from Jurkat T cells or HEK 293T cells). Cell lysates (50 μg of total protein) or immune complexes were separated by SDS-PAGE and transferred to nitrocellulose. Western blots were conducted with the indicated antibodies and developed with the appropriate horseradish peroxidase-conjugated secondary antibodies (Dianova) and the Luminol detection system (Carl Roth). The purified GST and GST-fusion protein of the cytoplasmic domain of CD18 were used in GST-pull-down experiments as previously described (51). Briefly, GST fusion proteins bound to glutathione-sepharose were incubated with lysates from HEK 293T cells expressing dsRed, dsRed-tagged FLNa or its mutants for 2 h at 4°C. Beads were washed and analyzed by Western blotting. For quantification, the intensity of the detected bands was calculated using the Kodak Image station 2000R (ID image software).

### *In vitro* Kinase Assay and the Phosphorylation Specificity for Ndr Kinases

For *in vitro* kinase assays, Ndr2- or FLAG-immunoprecipitates were divided into two equal aliquots. Both samples were pelleted and one of each paired sample was used for Western blotting (as described above). The remaining samples were washed three times and suspended in kinase buffer [25 mM 4-(2-hydroxyethyl)-1-piperazineethanesulfonic acid (HEPES pH7.4), 10 mM MgCl_2_, 1 mM dithiothreitol (DTT), and 100 μM [γ-^32^P]ATP (~1 μCi/μl, Perkin Elmer) or 100 μM ATP (Sigma Aldrich)] supplemented with either 5 μg myelin basic protein (MBP; Calbiochem) or with 1 μg purified GST-FLNa 19–24 Igl-repeats fusion protein and incubated for 30 min at 30°C. Reactions were stopped by addition of 5x sample buffer and resolved by SDS-PAGE. Gels were either dried and exposed to autoradiography films or were used for Western blotting (see above). For the characterization of Ndr2 or Ndr1 substrate specificity, FLAG-tagged Ndr/GFP-Mob2 complexes were isolated as previously described (42). Kinase reactions using the positional peptide library were performed as previously described (52). Soluble FLAG-tagged Ndr/GFP-Mob2 was incubated with [γ-^32^P]ATP (50 μM, 0.025 μCi/μl) and 198 unique peptide pools (50 μM) for 2 h at 30°C. Peptides had the general sequence Y-A-x-x-x-x-x-S/T-x-x-x-x-A-G-K-K(biotin), where “x” is an equimolar mixture of each of the 17 amino acids excluding Cys, Ser, and Thr. Each pool had one of the 20 amino acids fixed at one of the nine positions surrounding the central phosphoacceptor residue. Aliquots (2 μl) of each reaction were spotted onto streptavidin membranes (Promega) to capture the biotinylated peptides. The membranes were washed and exposed to a PhosphorImager as described (52). Data were quantified using ImageQuant software (Molecular Dynamics) and normalized so that the mean value for the 20 amino acids within a given position was 1; thus, values greater than 1 represent positive selections whereas values less than 1 represent negative selections. Heat maps were generated from average log_2_ transformed data from two independent runs using Microsoft Excel.

### Statistical Analysis

All data are presented as mean ± SEM. Statistical significance was determined between groups using a Student's *t*-test. A *p* ≤ 0.05 was considered statistically significant.

## Results

### Expression, Activation and Localization of Ndr2 in Primary Lymphocytes and T/B Cell Lines

**Figure 1:**
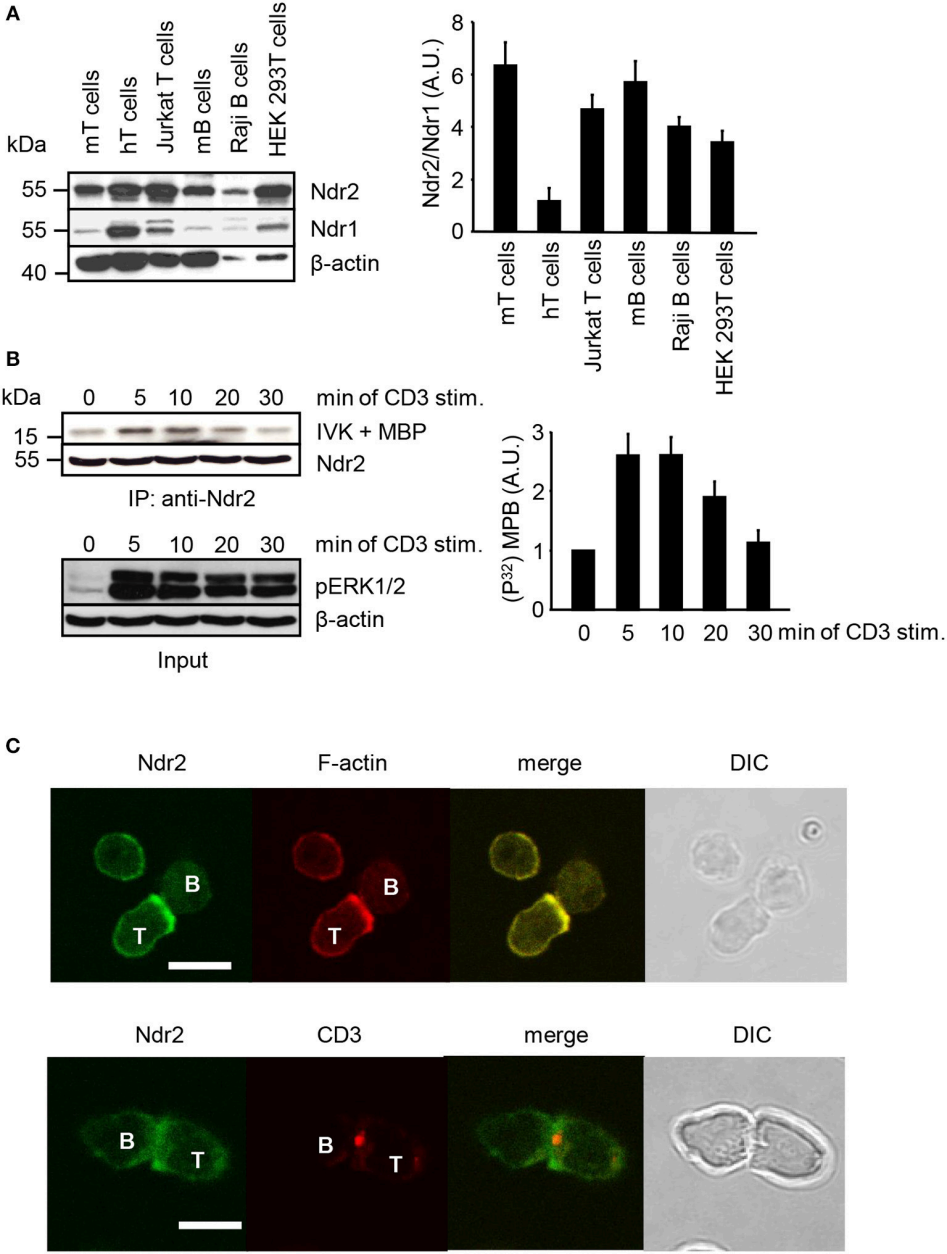
Expression profile, activation status and localization of Ndr2 in primary lymphocytes and lymphocyte-derived cell lines. **(A)** Total cell lysates of primary human and murine lymphocytes, Jurkat T cells, Raji B cells and HEK 293T cells were analyzed by Western blotting for expression of Ndr2 and Ndr1. β-actin staining served as loading control. Densitrometric analysis was performed to determine the Ndr2/Ndr1 ratio (*n* = 3; right graph). **(B)** Jurkat T cells were stimulated with CD3 antibodies for the indicated time points. Cells were lysed and Ndr2 was immunoprecipitated using Ndr2 rabbit antibody. Ndr2-precipitates were divided and one half of the precipitates was used to assess Ndr2 kinase activity by an *in vitro* kinase assay (IVK) using the myelin basic protein (MBP) as substrate. Phosphorylation of MBP was visualized with autoradiography. Densitrometric analysis were performed to determine the intensity of all MBP bands and values of MBP intensities from time point 0 min were set to 1 (*n* = 2; right graph). The second half of precipitates was used to detect Ndr2 by Western blotting. Aliquots of whole-cell extracts were analyzed for the phosphorylation status of ERK1/2 to verify successful stimulation of T cells (Input/lower panel). **(C)** Splenic B cells were loaded with OVA-peptide and co-incubated with purified T cells derived from OVA-TCR transgenic DO11.10 mice for 30 min. Cells were fixed, permeabilized and stained with an anti-Ndr2 Abs in combination with anti-rabbit IgG-FITC (green). F-actin was visualized with TRITC-Phalloidin (red) (upper panel). T/B cell conjugates were stained with Cy3-labeled anti-CD3 mAbs (red) and for Ndr2 (green; as described above; lower panel). Cells were imaged by confocal microscopy. Representative conjugates are shown. Each study was repeated at least three times and more than 25 conjugates were examined per condition. Scale bars define 5 μm. (mean ± SEM).

Western blot analysis was performed to assess the expression of Ndr2 in primary lymphocytes, T/B cell lines and HEK 293T cells. As depicted in Figure 1A, Ndr2 is expressed in primary human and murine lymphocytes, in the T and B cell lines (JE6.1 and Raji, respectively) and in HEK 293T cells. In contrast to human primary T cells, which expressed similar levels of Ndr2 and Ndr1, lower expression levels of Ndr1 were detected in primary murine lymphocytes, T/B cell lines, and HEK 293T cells. We next investigated whether TCR stimulation of Jurkat T cells activates endogenous Ndr2 (using an anti-Ndr2 antibody that specifically precipitates Ndr2 but not Ndr1, Figure S1). Kinase activity was assessed by an *in vitro* kinase assay using immunoprecipitated Ndr2 from non-activated and CD3-stimulated Jurkat T cells and myelin basic protein (MBP) as substrate (42). An increased phosphorylation of MBP was detected upon 5 min of TCR stimulation. Thereafter Ndr2 activity declined and reached baseline levels at 30 min after stimulation (Figure 1B, upper and left panels). In contrast to Ndr2 activity, ERK1/2 phosphorylation persisted for 30 min, thus verifying proper T-cell stimulation (Figure 1B, lower panel). In addition to TCR-mediated activation of Ndr2, we studied the localization of Ndr2 at the immunological synapse (IS). To this end, CD4^+^ T cells were isolated from ovalbumin-TCR transgenic mice (DO11.10). IS formation was induced by co-incubation with OVA peptide-loaded *ex vivo* isolated B cells. The T/B cell-pairs were assessed by confocal microscopy. As depicted in Figure 1C, Ndr2 was recruited to the immunological synapse, where it co-localized with F-actin (85% ± 7.48 of all analyzed T/B cell-pairs), but not with CD3. This indicates that Ndr2 is localized at the peripheral part of the IS. In summary, the data shown in Figure 1 indicate (i) that Ndr2 is expressed in lymphocytes, (ii) that it is rapidly and transiently activated upon TCR-stimulation and (iii) recruited to the cytoskeleton-enriched region of the IS.

### Kinase Activity of Ndr2 Is Required For TCR-Mediated Adhesion, Interaction With APCs and LFA-1 Activation

**Figure 2:**
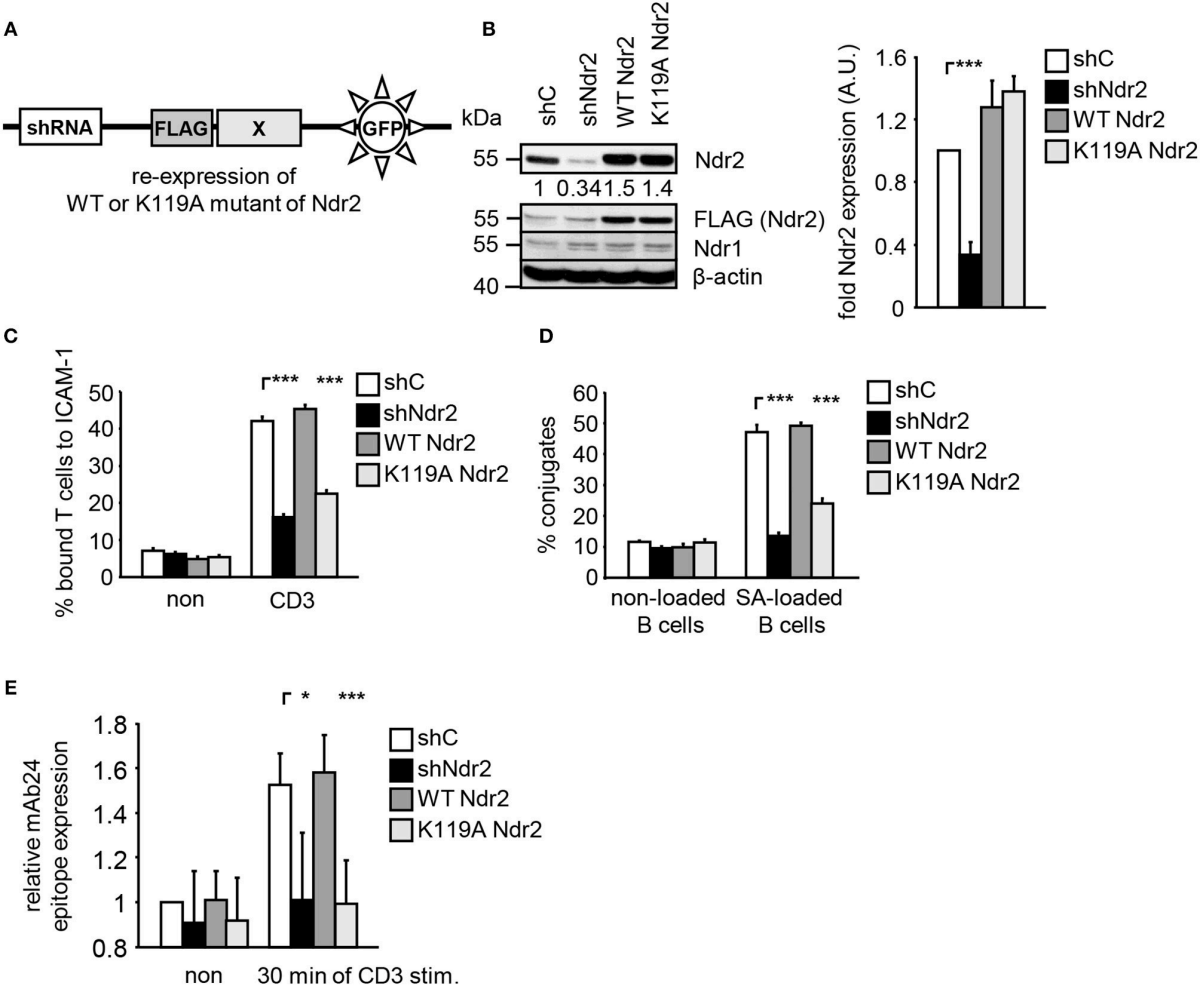
Kinase activity of Ndr2 controls TCR-mediated adhesion, interaction of T cells with APCs and LFA-1 activation. **(A)** Schematic representation of the suppression/re-expression plasmids for Ndr2 used in this study. **(B)** Jurkat T cells were transfected with suppression/re-expression plasmids which do not suppress endogenous Ndr2 (shC), reduce the endogenous protein level of Ndr2 (shNdr2), re-express a FLAG-tagged shRNA-resistant wild type Ndr2 (WT Ndr2) or re-express its kinase dead mutant (K119A Ndr2). 48 h after transfection, lysates were analyzed by Western blotting for Ndr2, Ndr1, FLAG, and β-actin (loading control). Numbers represent the reduction and re-expression of Ndr2 and its mutant after normalization to the Ndr2 expression level of the shC-tranfected control cells, which were set to 1 (*n* = 4; right graph). **(C)** Transfected Jurkat T cells as described in **(B)** were analyzed for their ability to adhere to ICAM-1-coated wells in a resting state or stimulated for 30 min with CD3 antibodies. Adherent cells were counted and calculated as percentage of input (*n* = 4). **(D)** Cells were transfected as described in **(B)** and analyzed for their ability to form conjugates with DDAO-SE (red)-stained Raji B cells that were pulsed without (non) or with superantigen (SA) for 30 min. The percentage of conjugates was defined as the number of double positive events in the upper right quadrant (*n* = 4). **(E)** Jurkat T cells transfected as described in **(B)** were left untreated (non) or stimulated with CD3 antibodies (CD3), followed by staining with the anti-LFA-1 antibody mAb24 which recognizes the high affinity conformation of LFA-1. mAb24 epitope expression was assessed by flow cytometry within the GFP gate and data are normalized against LFA-1 expression detected by MEM48 (*n* = 4). (mean ± SEM; *p ≤ 0.05; ***p ≤ 0.001).

To address the functional importance of Ndr2 for TCR-mediated LFA-1 signaling, we generated suppression/re-expression plasmids that knock down endogenous Ndr2 and simultaneously re-express FLAG-tagged shRNA-resistant wild type (WT Ndr2) or FLAG-tagged kinase dead mutant of Ndr2, carring a mutation of lysine 119 to alanine [K119A Ndr2 (42)] (Figure 2A). As shown in Figure 2B, the suppression construct (shNdr2) strongly reduced endogenous Ndr2 levels when compared to vector control levels (shC). In addition, the suppression/re-expression plasmids allowed proper re-expression of either WT or K119A mutant of Ndr2, respectively. Importantly, suppression of Ndr2 and re-expression of Ndr2-forms did not alter the endogenous expression levels of Ndr1 (Figure 2B). As depicted in Figures 2C,D, downregulation of Ndr2 in Jurkat T cells diminished their ability to adhere to ICAM-1 (Figure 2C) and to interact with superantigen-pulsed B cells (Figure 2D). Re-expression of the shRNA-resistant WT Ndr2 rescued the ability of the cells to adhere to ICAM-1 and to interact with APCs (Figures 2C,D). In contrast to WT Ndr2 re-expressing cells, re-expression of the K119A mutant of Ndr2 did not rescue TCR-mediated adhesion to ICAM-1 or interaction with superantigen-pulsed B cells (Figures 2C,D). Of note, the attenuated T-cell adhesion and interaction with APCs were not due to altered expression levels of the TCR or LFA-1 (Figure S3A).

To investigate whether Ndr2 affects the TCR-induced affinity state of LFA-1, we took advantage of the conformation-specific antibody mAb24. This antibody specifically binds only the high affinity conformation of LFA-1, which is required for ligand binding (1). Indeed, suppression of Ndr2 in T cells interfered with the TCR-mediated induction of the mAb24 epitope within LFA-1 compared to shC-transfected cells (Figure 2E). Re-expression of WT Ndr2 rescued the ability of mAb24-binding to LFA-1 in response to TCR stimulation whereas this was not the case in cells re-expressing the K119A mutant of Ndr2 (Figure 2E). In summary, these findings suggest that TCR-mediated adhesion, interaction of T cells with APCs and affinity modulation of LFA-1 require enzymatically active Ndr2 in Jurkat T cells.

### Ndr2 Phosphorylates FLNa at Serine 2152

**Figure 3:**
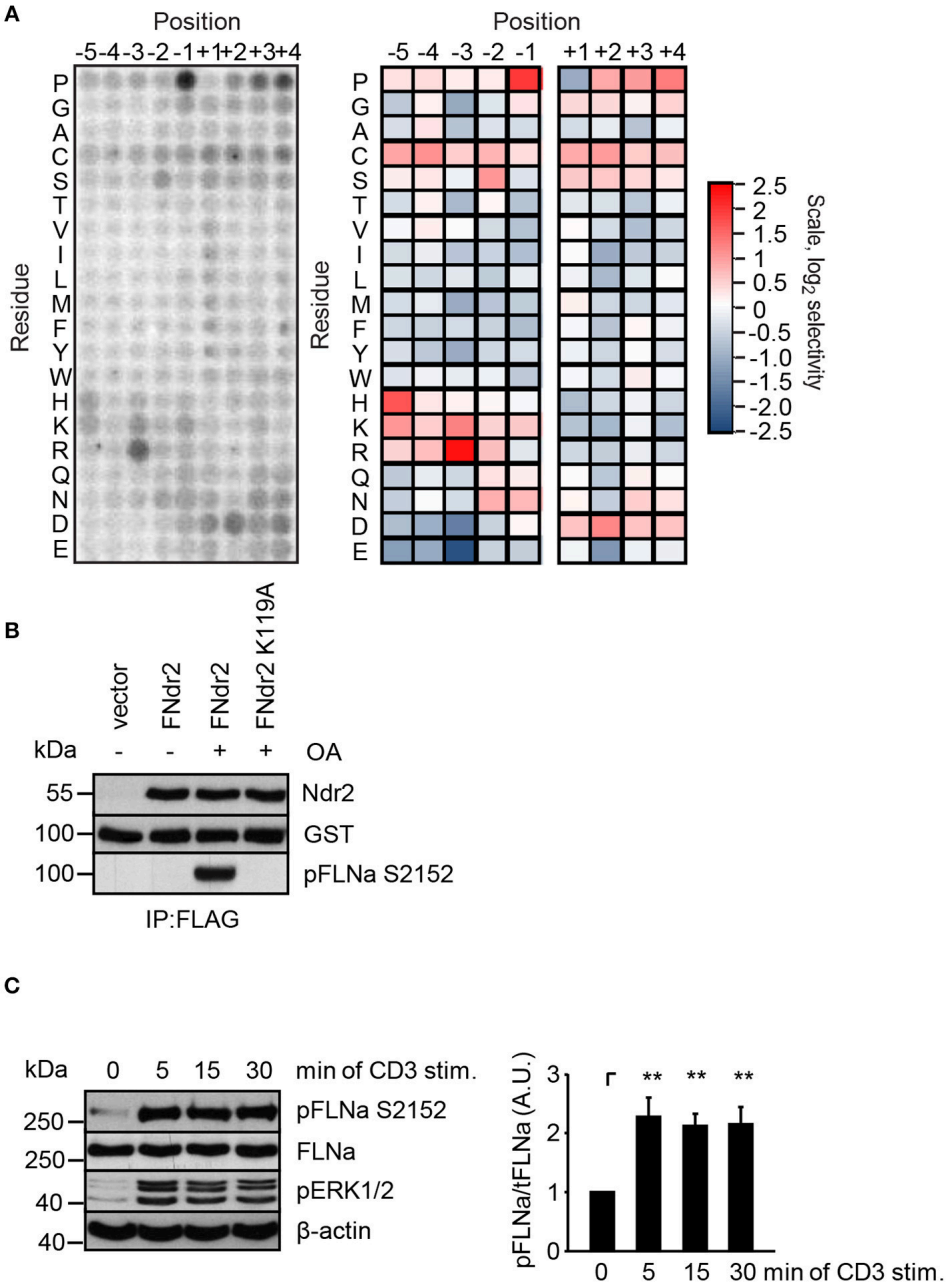
Ndr2 phosphorylates FLNa at S2152 *in vitro*. **(A)** Purified WT Ndr2/Mob2 heterodimer was used to phosphorylate a positional scanning peptide library using radiolabeled ATP. The degree of phosphorylation of each component of the library, harboring the indicated amino acid residue at the indicated position relative to the phosphorylation site, is shown at left. Quantified data were normalized, log_2_ transformed, and used to generate a heat map shown at right (*n* = 2). **(B)** HEK 293T cells were transfected with either empty pEFBOS vector (vector) or plasmids encoding FLAG-tagged wild type Ndr2 (FNdr2) and a kinase dead (K119A) mutant of Ndr2 (FNdr2K119A). Cells were left untreated or treated with okadaic acid (OA), lysed and Ndr2 was immunoprecipitated using FLAG antibodies. A GST-FLNa fragment (19–24 repeats) was used as substrate for an *in vitro* kinase assay. Reactions were analyzed by Western blotting with the indicated antibodies (*n* = 3). **(C)** Jurkat T cells were left untreated or stimulated for the indicated time points with CD3 antibodies. Cells were lysed and analyzed by Western Blotting with the indicated antibodies. Aliquots of whole-cell extracts were analyzed for the phosphorylation status of ERK1/2 to verify successful stimulation of T cells. Densitrometric analysis of the FLNa phosphorylation status at Serine 2152 (pFLNa) normalized to total FLNa (tFLNa) (*n* = 4) (mean ± SEM; **p ≤ 0.01).

Having shown that enzymatically active Ndr2 is required for TCR-induced affinity modulation of LFA-1 (Figure 2E) we next asked which integrin-activity modulator might be controlled by Ndr2. To this end, we first determined the amino acid motifs phosphorylated by Ndr2, using FLAG-tagged Ndr2/GFP-Mob2 complex from HEK 293T cells and a positional peptide library as previously described (42, 52). Using this approach we identified an R-X-P-S/T motif as an optimal sequence for Ndr2 kinase (Figure 3A). A qualitatively similar sequence preference was observed for the Ndr1-Mob2 complex (Figure S4B). Reactions performed with catalytically-inactive FLAG-tagged Ndr2 mutant (K119A)/GFP-Mob2 exhibited negligible kinase activity (Figure S4A), demonstrating that the radiolabeled peptides were not phosphorylated by a contaminating kinase that accidently was co-purified with Ndr2. The elevated signals for serine residues at multiple positions are likely due to the presence of two potential phosphoacceptors within the peptide. Threonine signals were not similarly elevated, suggesting that Ndr2 prefers to phosphorylate serine residues over threonine, at least *in vitro*. This preference was also reflected in the relative phosphorylation by Ndr2 of peptides having either serine or threonine as the sole phosphoacceptor residue [personal communication by J. L. Johnson and L. Cantley (Meyer Cancer Center, Weill Cornell Medicine College, New York, USA)].

Using integrin-activity modulation as a criterion, an online protein database search revealed that FLNa, a previously described negative regulator of LFA-1 activation (5, 6), contains a suitable “RXPS” motif at the serine 2152 (S2152). Since phosphorylation of this motif has been shown to reduce focal adhesion formation (53) we further investigated the FLNa S2152 phosphorylation site as a putative target of Ndr2 kinase. In order to address whether Ndr2 directly phosphorylates FLNa at S2152, we performed an *in vitro* kinase assay using FLAG-tagged wild type Ndr2 immunoprecipitated from HEK 293T cells (42). Prior to immunoprecipitation of Ndr2, transfected cells were left untreated or stimulated with okadeic acid (OA) for 1 h, a phosphatase inhibitor and known activator of Ndr2 (42). Using the GST-fusion protein of a C-terminal fragment of FLNa (immunoglobulin-like (Igl) repeats 19–24 of human FLNa) containing the S2152 phosphorylation site as substrate, we detected S2152-specific phosphorylation after OA-stimulation, but not in untreated FLAG-tagged wild type Ndr2 expressing cells (Figure 3B). In contrast, a K119 mutant of Ndr2 precipitated from OA-stimulated cells did not phosphorylate FLNa at S2152 (Figure 3B) excluding an unspecific FLNa S2152 phosphorylation effect by a co-precipitated kinases. These data suggest that Ndr2 is able to phosphorylate FLNa at S2152 *in vitro*.

**Figure 4:**
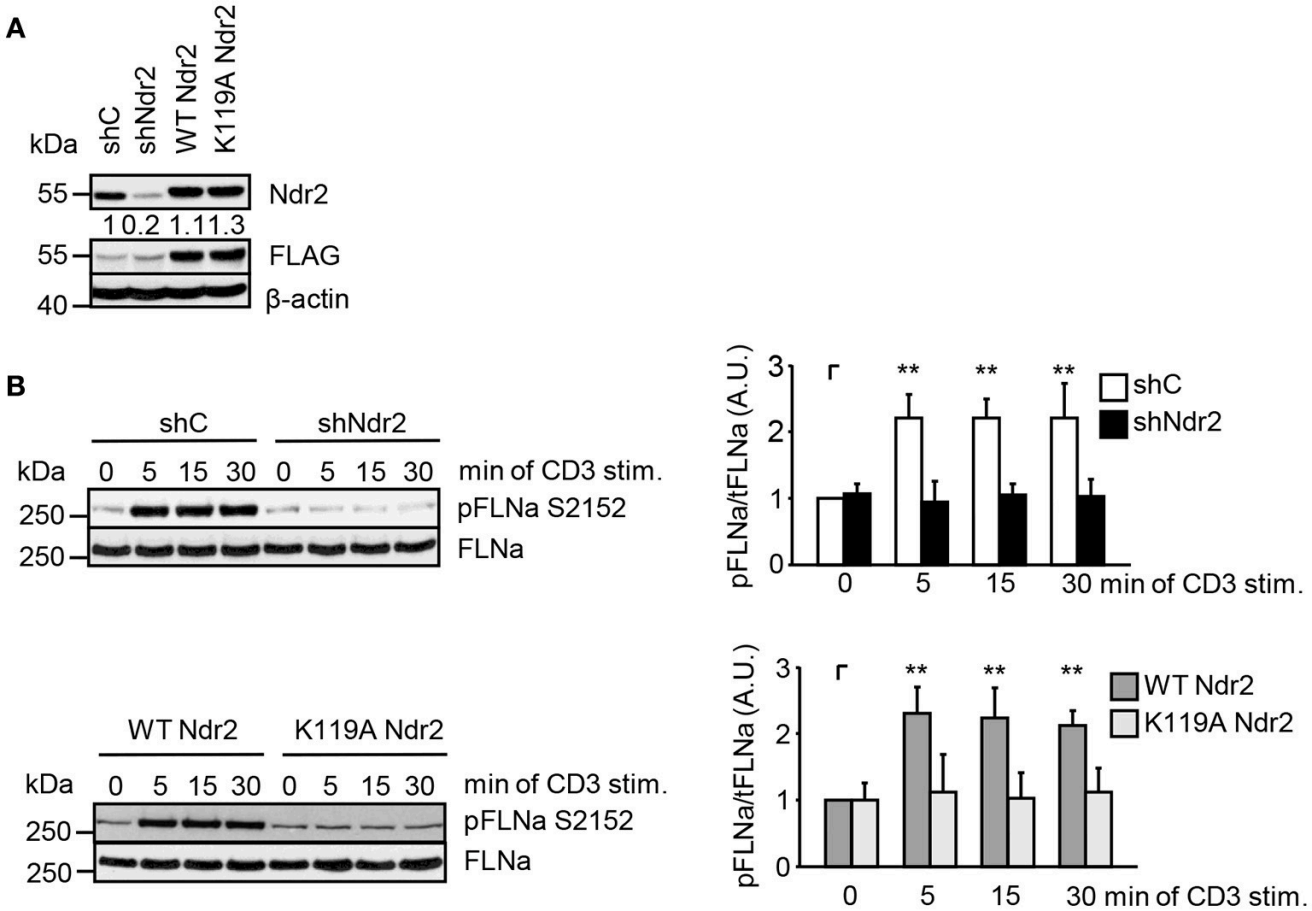
Ndr2 phosphorylates FLNa at S2152 in Jurkat T cells *in vivo*. **(A)** Jurkat T cells were transfected with suppression/re-expression constructs which suppress endogenous Ndr2 (shNdr2) and re-express a FLAG-tagged shRNA-resistant wild type (WT Ndr2) or a kinase-dead mutant of Ndr2 (K119A Ndr2). Numbers represent the reduction and re-expression of Ndr2 and its mutant after normalization to the Ndr2 expression level of the shC-tranfected control cells. **(B)** At 48 h post-transfection, cells left untreated or stimulated for the indicated time points with CD3 antibodies. Cells were lysed and analyzed by Western Blotting with the indicated antibodies. Densitrometric quantification of FLNa phosphorylation at Serine 2152 (pFLNa) normalized to total FLNa (tFLNa) (*n* = 3). (mean ± SEM; **p ≤ 0.05).

Next, we analyzed the phosphorylation status of FLNa at S2152 upon TCR-triggering in Jurkat T cells. As depicted in Figure 3C, TCR-stimulation induced the phosphorylation of FLNa at S2152, which peaked at 5 min and remained at this level for 30 min. Note that the expression levels of FLNa were not changed upon TCR-stimulation (Figure 3C). Having shown that TCR-stimulation induces phosphorylation of FLNa at S2152, we examined whether loss of Ndr2 or its kinase activity would alter S2152 phosphorylation of FLNa. As shown in Figures 4A,B, silencing of Ndr2 expression in Jurkat T cells strongly reduced S2152 phosphorylation of FLNa upon TCR stimulation. Re-expression of WT Ndr2 rescued the phosphorylation status of FLNa at S2152 to a similar extent as found for shC-transfected cells. In contrast to wild type Ndr2 expressing cells, re-expression of the K119A mutant of Ndr2 attenuated TCR-mediated S2152 phosphorylation of FLNa (Figures 4A,B). Taken together the data depicted in Figures 3 and 4 indicate that Ndr2 phosphorylates FLNa at S2152 both *in vitro* and *in vivo*.

**Figure 5:**
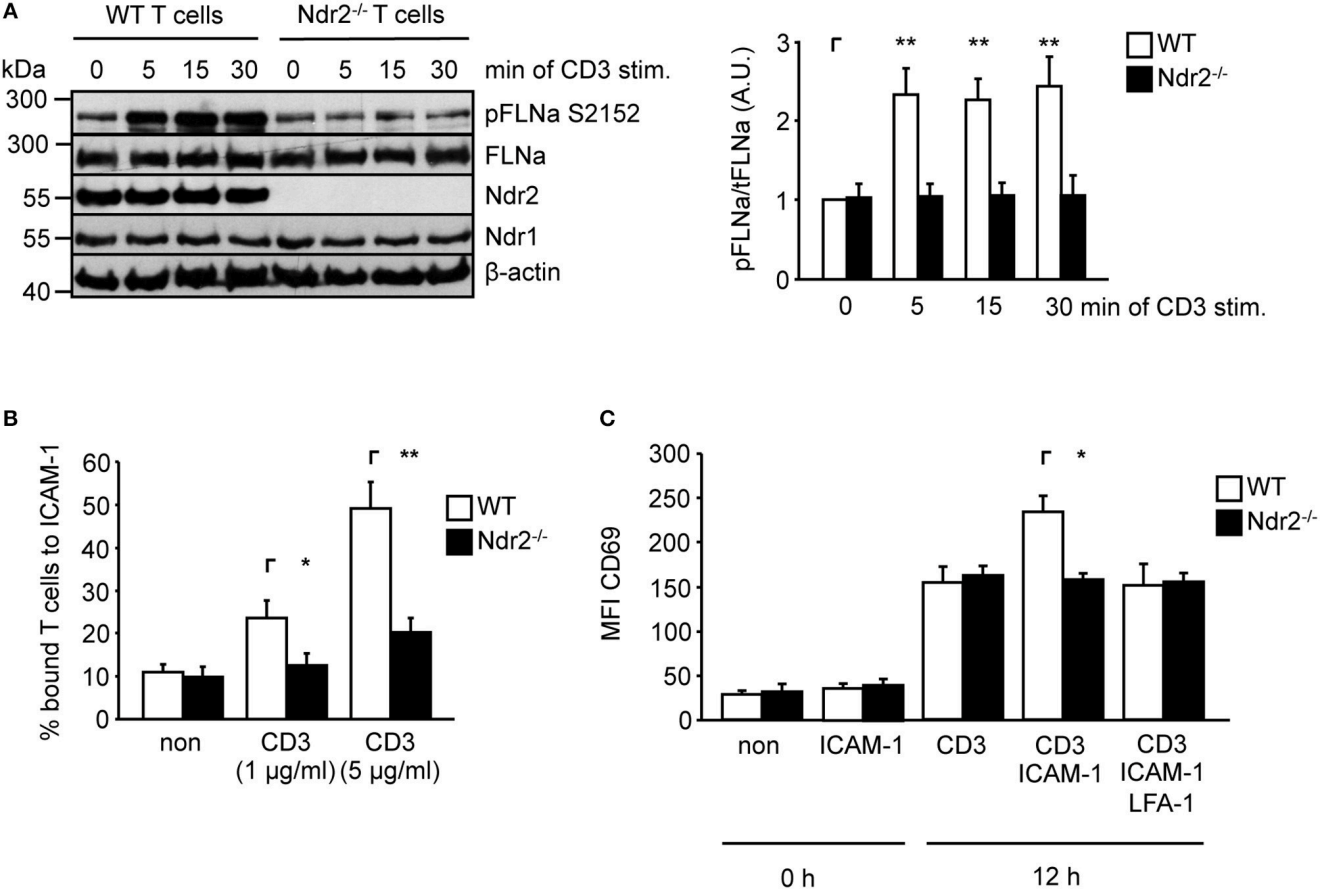
Ndr2-deficiency in murine CD4^+^ T cells attenuates TCR-induced FLNa phosphorylation at S2152, T-cell adhesion and LFA-1-dependent upregulation of CD69 *in vitro*. **(A)** Purified splenic wild type (WT) and Ndr2^−/−^ CD4^+^ T cells were left untreated or stimulated with anti-CD3 antibodies for the indicated time points. Lysates were prepared and analyzed by Western blotting using the indicated antibodies. Densitrometric quantification of FLNa phosphorylation at Serine 2152 (pFLNa) normalized to total FLNa (tFLNa) (*n* = 3). **(B)** Purified splenic WT and Ndr2^−/−^ CD4^+^ T cells were left untreated (non) or stimulated with anti-CD3 antibodies (CD3) and subsequently analyzed for their ability to bind plate-bound Fc-ICAM-1. Adherent cells were counted and calculated as percentage of input (*n* = 3). **(C)** Purified splenic CD4^+^ T cells from WT and Ndr2 mice were cultured with plate-bound anti-CD3 antibodies (CD3) in the absence or presence of Fc-ICAM-1 (ICAM-1) with or without blocking LFA-1 antibodies (LFA-1) for 12 h. The upregulation of the activation marker CD69 of unstimulated (0 h) or activated T cells (12 h) were assessed by flow cytometry to determine the mean fluorescence intensity (MFI) (*n* = 3). (mean ± SEM; *p ≤ 0.05, **p ≤ 0.01).

To verify whether Ndr2-deficiency attenuates TCR-mediated phosphorylation of FLNa in murine T cells, we isolated CD4^+^ T cells from wild type and Ndr2^−/−^ knockout mice (40). Note that in line with previously published data by Tang et al. (36), loss of Ndr2 in mice did not affect thymic development or CD4^+^ and CD8^+^ T-cell distribution in the spleen and lymph node (data not shown). As shown in Figure 5A, TCR-stimulation induced the phosphorylation of FLNa at S2152 in wild type CD4^+^ T cells with a similar phosphorylation kinetic in shC-transfected Jurkat T cells which peaked at 5 min and remained at this level for 30 min. In contrast to wild type CD4^+^ T cells, TCR-induced S2152 phosphorylation of FLNa was abrogated in Ndr2^−/−^ CD4^+^ T cells. Similar to downregulation of Ndr2 in Jurkat T cells, Ndr2^−/−^ CD4^+^ T cells showed diminished ability to adhere to ICAM-1 (Figure 5B). Importantly, the attenuated TCR-mediated phosphorylation of FLNa and T-cell adhesion of Ndr2^−/−^ CD4^+^ T cells were not due to altered expression levels of FLNa, the TCR (CD3) or LFA-1 (Figure S3C).

ICAM-1-binding to the high affinity conformation of LFA-1 in T cells induces co-stimulatory signaling pathway(s) that enhances the TCR-mediated CD69 upregulation (54, 55). Hence we analyzed the upregulation of CD69 upon TCR stimulation in the absence or presence of plate-bound ICAM-1. As depicted in Figure 5C the upregulation of this activation marker was significant increased in the presence of TCR/ICAM-1 in WT CD4^+^ T cells. The addition of a LFA-1 blocking Ab to these cells reduced the CD69 expression to similar levels as detected for TCR-stimulation alone, showing that these processes were indeed LFA-1 dependent. In contrast to WT CD4^+^ T cells, loss of Ndr2 did not lead to an enhanced TCR/ICAM-1-induced expression of this marker compared to WT T cells. The expression levels of CD69 was not different in Ndr2-deficient CD4^+^ T cells upon TCR- stimulation in the absence or presence ICAM-1 and moreover comparable with TCR-stimulated WT T cells (Figure 5C). Together, the data presented in Figure 5 reveal a crucial role of Ndr2 for TCR-induced inside-out and outside-in signaling.

### Overexpression of FLNa and Its S2152A Mutant Attenuates TCR-Mediated LFA-Activation

**Figure 6:**
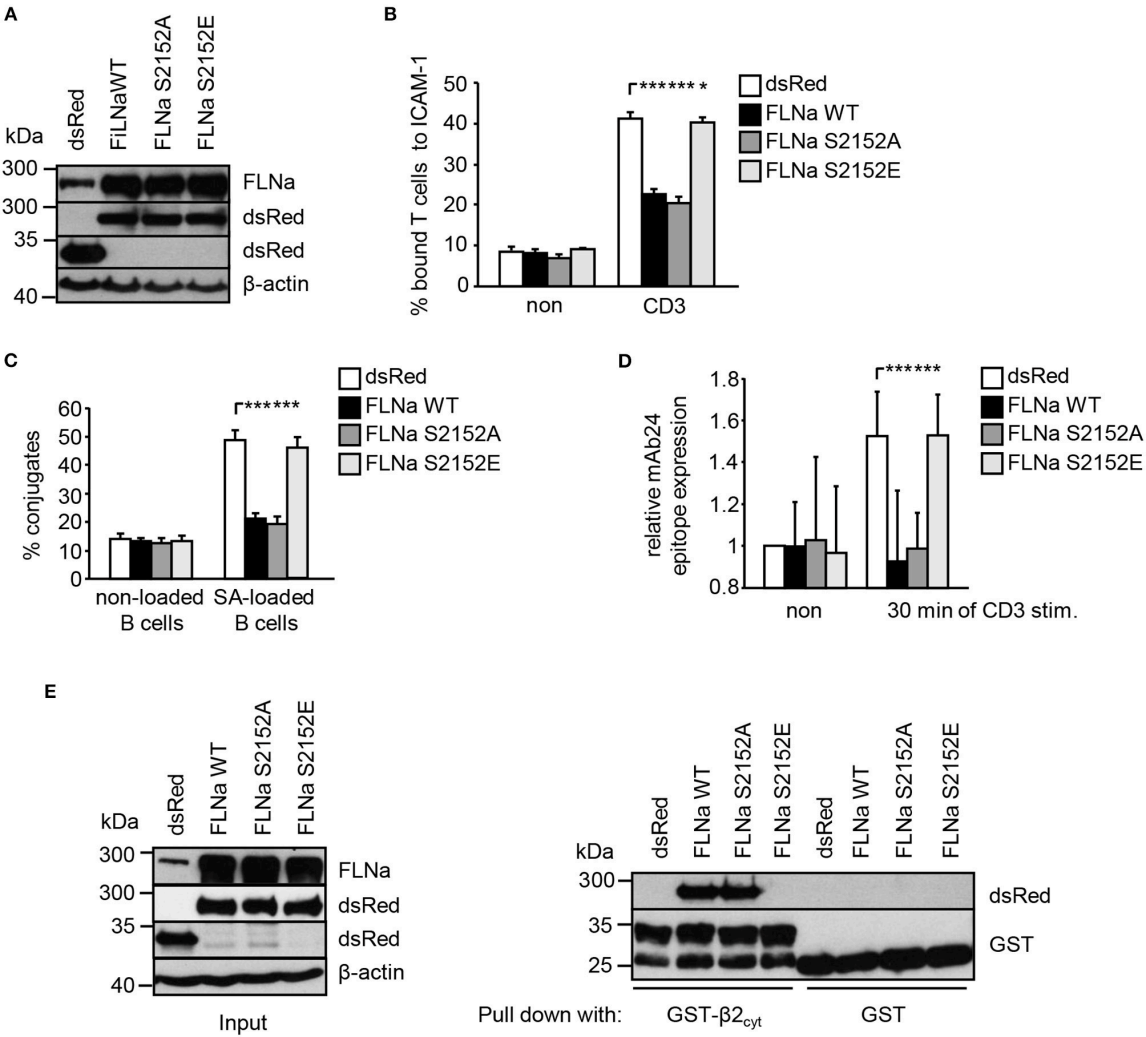
Mutation of S2152A within FLNa abolishes TCR-mediated T-cell adhesion, interaction with APCs and LFA-1 activation. **(A)** Jurkat T cells were transiently transfected with dsRed C1 vector (dsRed) or plasmids encoding wild type (WT) dsRed-tagged FLNa (FLNa WT) or dsRed-tagged S2152A or dsRed-tagged S2152E FLNa mutants (FLNa S215A and FLNa S2152E). After 24 h the expression of WT and its mutants were analyzed by anti-dsRed and FLNa immunoblotting. Detection of β-actin served as loading control. **(B)** Jurkat T cells transfected as described in **(A)** were left untreated (non) or stimulated for 30 min with CD3 antibodies. Cells were analyzed for adhesion to ICAM-1-coated 96 well plates. Bound cells were counted and calculated as percentage of input (*n* = 4) (mean ± SEM; *p ≤ 0.05, ***p ≤ 0.001). **(C)** Cells were transfected as described in **(A)** and analyzed for their ability to form conjugates with DDAO-SE (red)-stained Raji B cells that were pulsed without (non) or with superantigen (SA) for 30 min at 37°C. The percentage of conjugates was defined as the number of double-positive events in the upper right quadrant (*n* = 4) (mean ± SEM; ***p ≤ 0.001). **(D)** Jurkat T cells transfected as described in **(A)** were left untreated (non) or stimulated with anti-CD3 antibodies (CD3), followed by staining with the anti-LFA-1 antibody mAb24 to detect the high affinity conformation of LFA-1. mAb24 epitope expression was assessed by flow cytometry and data are normalized against LFA-1 expression detected by MEM48 (*n* = 4) (mean ± SEM; ***p ≤ 0.001). **(E)** HEK 293T cells were transfected with either dsRed, dsRed-tagged FLNa wild type (FLAa WT) or its mutants (FLNa S2152A and FLNa S2152E). 24 h after transfection, whole cell extracts were prepared and analyzed for the expression of dsRed and dsRed-tagged FLNa forms by Western blotting using the indicated antibodies (left panel). Lysates were incubated with GST-fusion proteins bound to glutathione-sepharose beads. Precipitates were analyzed by Western blotting using the indicated antibodies (right panel). One representative experiment of 3 is shown. (mean ± SEM; *p ≤ 0.05, ***p ≤ 0.001).

Previously it had been shown that loss of FLNa induces spontaneous LFA-1 activation in the non-stimulated pro-B-cell line BAF (6). In line with this study, suppression of FLNa expression in Jurkat T cells (Figure S5A) induced constitutive and enhanced TCR-stimulated adhesion to ICAM-1, interaction with APCs and LFA-1 affinity modulation (Figures S5B–D), while not affecting the expression of the TCR or LFA-1 (Figure S5E). We speculated that overexpression of wild type FLNa would impede TCR-mediated adhesion and T-cell interaction with APCs. Indeed, Jurkat T cells overexpressing WT FLNa showed approx. 50% decreased adhesion to ICAM-1 compared to control cells upon TCR-stimulation (Figures 6A,B). Similarly, WT FLNa expressing Jurkat T cells showed an attenuated capability to interact with superantigen-loaded B cells (Figure 6C). In line with these observations, we found that cells overexpressing the dsRed-tagged FLNa S2152A mutant (mimics loss of phosphorylation S2152) attenuated TCR-mediated adhesion of Jurkat T cells to ICAM-1 (Figure 6B) and the interaction with APCs (Figure 6C). In addition, both overexpressing WT FLNa and its S2152A mutant in Jurkat T cells completely blocked the induction of the high affinity conformation of LFA-1 upon TCR stimulation (Figure 6D). Flow cytometric analysis revealed comparable levels of CD18 and the TCR on the surface of these FLNa WT or FLNa S2152A expressing Jurkat T cells (Figure S3B).

In contrast to WT FLNa and the S2152A mutant, Jurkat T cells expressing a S2152E mutant of FLNa (mimics phosphorylation of S2152) showed similar levels of TCR-mediated adhesion, interaction with APCs and LFA-1 activation compared to dsRed expressing cells. These data suggest that both WT and the S2152A mutant of FLNa act in a dominant negative fashion for TCR-mediated LFA-1 activation.

Given the above data, we speculated that in contrast to FLNa S2152E, WT FLNa and its S2152A mutant still interact with the cytoplasmic region of CD18. To answer this question, we transfected HEK293T cells with dsRed only, dsRed-tagged FLNa WT and its two S2152A vs. S2152E mutants. Cell lysates were used for precipitation using either purified GST or GST-CD18_cyt_, respectively. Using this approach we detected an association of the GST-CD18_cyt_ fusion protein only with WT [in line with previously published data (6)] and S2152A FLNa but not with the S2152E FLNa mutant (Figure 6E). These data indicate that the phosphorylation of S2152 of FLNa attenuates the interaction with the cytoplasmatic region of the β2-chain of LFA-1.

### Activated Ndr2 Promotes FLNa Release From LFA-1

**Figure 7:**
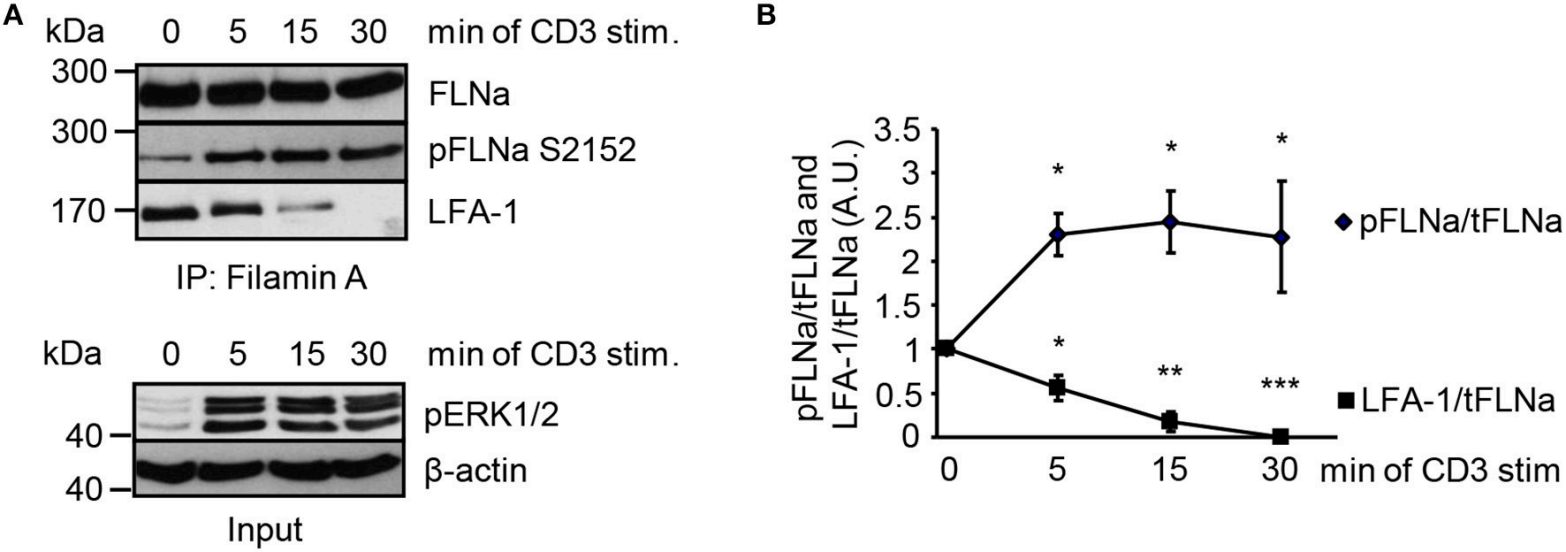
TCR-stimulation of Jurkat T cells releases LFA-1 from FLNa. **(A)** Jurkat T cells were left untreated or stimulated for the indicated time points with CD3 antibodies. Lysates were prepared and used for immunoprecipitation of FLNa. Precipitates were analyzed by Western blotting for the phosphorylation status of FLNa at S2152 and LFA-1 association. Aliquots of whole-cell extracts were analyzed for the phosphorylation status of ERK1/2 to verify successful stimulation of T cells (lower panel; Input). **(B)** Densitrometric analysis of the FLNa phosphorylation status at Serine 2152 (pFLNa) normalized to precipitated total FLNa (tFLNa) or of LFA-1 to precipitated total FLNa (tFLNa) (*n* = 4). (mean ± SEM; *p ≤ 0.05, **p ≤ 0.01, ***p ≤ 0.001).

In non-activated T cells, FLNa is bound to LFA-1 and stabilizes its closed conformation (6). We hypothesized that stimulation of the TCR releases LFA-1 from FLNa and further that, this event correlates with an increased phosphorylation status of FLNa at S2152. To test this idea, we immunoprecipitated FLNa from non-stimulated and TCR-stimulated Jurkat T cells. In resting Jurkat T cells, we detected an association of FLNa with LFA-1 (Figures 7A,B). Triggering of the TCR induced rapid LFA-1 dissociation from FLNa which correlated with increased phosphorylation of S2152 (Figures 7A,B).

**Figure 8:**
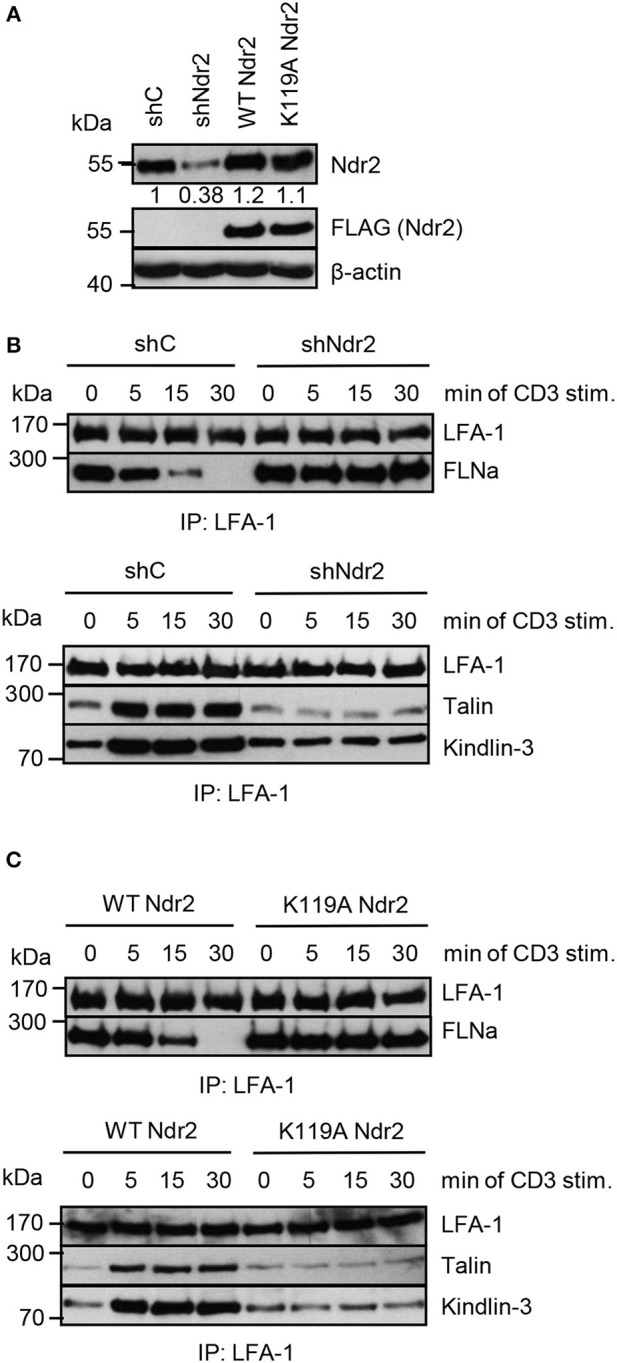
Activated Ndr2 releases FLNa binding from LFA-1. **(A)** Jurkat T cells were transfected with constructs that suppress endogenous Ndr2 (shNdr2) and re-express a FLAG-tagged shRNA-resistant wild type (WT Ndr2) or a kinase-dead mutant of Ndr2 (K119A Ndr2). 48 h after transfection, whole-cell extracts were prepared and analyzed by Western blotting using the indicated antibodies. Numbers represent the reduction and re-expression of Ndr2 and its mutant after normalization to the Ndr2 expression level of shC-tranfected control cells. **(B,C)** Cells left untreated or stimulated for the indicated time points with CD3 antibodies. Lysates were used for immunoprecipitation of LFA-1 using anti-CD11a antibodies. Precipitates were divided and analyzed by Western blotting for FLNa, Talin and Kindlin-3 association. Densitrometric analyses of FLNa, Talin, or Kindlin-3 associated to LFA-1 are depicted in Figure S8.

Next, we assessed whether the Ndr2-mediated phosphorylation of FLNa at S2152 releases FLNa from LFA-1 and thus allowing binding of the two integrin activating proteins Talin and Kindlin-3 to LFA-1 and promote LFA-1 activation. To test this hypothesis, we immunoprecipitated LFA-1 from non-activated and TCR-stimulated Jurkat T cells and assessed binding of Talin and Kindlin-3, respectively. In resting shC-transfected Jurkat T cells, we detected an association of FLNa with LFA-1, whereas Talin or Kindlin-3 interaction with LFA-1 was detectated only at low levels. Upon TCR-stimulation, FLNa association with LFA-1 declined within 30 min while Talin/Kindlin3 association to LFA-1 increased (Figures 8A,B). Importantly, downregulation of Ndr2 abrogated the TCR-inducible association of these two LFA-1 interacting proteins (Figure 8B). In contrast, in cells re-expressing the catalytically inactive mutant of Ndr2 (K119A Ndr2), TCR-stimulation did not induce the release of FLNa from LFA-1 and consequently the recruitment of Talin and Kindlin-3 to LFA-1 was blocked (Figure 8C). WT Ndr2 expressing cells by contrast showed similar FLNa disassociation dynamics as shC-transfected cells (Figure 8C). In summary our findings suggest that TCR-mediated activation of Ndr2 leads to phosphorylation of FLNa at S2152. This releases FLNa from LFA-1 and subsequently allows the association of Talin and Kindlin-3 thus inducing LFA-1 activation.

## Discussion

In this study we report that Ndr2 kinase activity is required for TCR-mediated LFA-1 affinity regulation to facilitate T-cell adhesion and interaction with APCs. Our data reveal that Ndr2 supports LFA-1 activation during T-cell activation by phosphorylating FLNa at S2152 and by triggering its release from the cytoplasmic chain of CD18.

Ndr kinases are highly conserved; however, cell-type specific expression levels as well as spatial and conformational constraints on activation and enzyme-substrate interactions may result in isoform specificity of the two mammalian Ndr isoforms Ndr1 and Ndr2. Here, we focused on the role of Ndr2 rather than on the previously studied Ndr1 (10), as we found a predominant expression of this isoform in Jurkat T cells as well as in murine T cells and because we observed its prominent recruitment to the IS upon TCR stimulation. Importantly, knockdown/knockout and/or re-expression of Ndr2 did not affect Ndr1 expression in Jurkat T cells, in line with our previous observations in the mouse brain (40).

Our data indicate that Ndr2 is activated within 5 min after TCR stimulation. However, how TCR-mediated Ndr1/2 activation is achieved in T cells remains unsolved. Activation of Ndr kinases generally requires the phosphorylation of the C-terminal hydrophobic motif (pThr442/444) through Mst1 and Mst3 kinases and N-terminal binding of the Mob protein as a co-activator (36, 42, 56). While the role of Mob proteins in T cells is still unclear, it has recently been shown that Ndr1 and/or Ndr2 are phosphorylated and activated in an Mst1/2-dependent manner upon chemokine and TCR-stimulation of murine T cells (10, 36). As depicted in Figure S6, we confirmed that loss of Ndr2 does not affect the phosphorylation status of either Mst1 nor Mst2 upon TCR-stimulation, suggesting that in Jurkat T cells Ndr2 is located downstream of Mst kinases and can be activated by these kinases upon TCR-stimulation.

In an unbiased screen we identified an RXPT/S motif as putative phosphorylation site for Ndr2. We demonstrated that the corresponding motif around serine 2152 of FLNa is a target site for Ndr2 kinase *in vitro* and *in vivo*. Serine 2152 within FLNa represents the main phosphorylation site as shown by several studies. Treatment of cells with various stimuli e.g., insulin (53), insulin-like growth factor-I (57), epidermal growth factor (58), arsenic (59), cAMP or agonists that increase intracellular cAMP levels (60–64) leads to S2152 phosphorylation of FLNa in epithelial, platelets, smooth muscle cells, cancer cells and fibroblasts, respectively. To our knowledge, this is the first report showing that TCR-stimulation also triggers the phosphorylation of FLNa at S2152.

Similar to TCR stimulation, triggering of the monocyte chemokine receptor 2 (CCR2) also leads to phosphorylation of FLNa at S2152 (65). Further, stimulation of the chemokine receptor CCR7 induces activation of Ndr kinases in thymocytes (36). CD4^+^ or CD8^+^ single positive thymocytes from Ndr1/2-double deficient mice are defective in response to CCL19 (ligand of CCR7)-induced transwell migration (36). Therefore, it is possible that Ndr kinases are also activated upon chemokine triggering to phosphorylate S2152 of FLNa, regulating adhesive and/or migratory response(s) of these T cells.

As depicted in Figure 6, our data reveal that overexpression of a S2152A mutant of FLNa attenuates TCR-mediated LFA-1 affinity modulation (to a similar extend then wild type FLNa) and subsequently leads to impaired T-cell adhesion and interaction with APCs. FLNa contains 24 immunoglobulin-like repeats (Igl-repeat), with a binding site for the cytoplasmic domain of the LFA-1 β2-chain in the Igl-repeat 21 (5) and the S2152 phosphorylation site in Igl-repeat 23. We observed that wild type FLNa [in line with previously published data (6)] and FLNa S2152A, but not the FLNa mutant that mimics phosphorylation (S2152E) interact with the GST-CD18_cyt_ fusion protein. These data suggest that Ndr2-mediated phosphorylation of S2152 residue on Ig1-repeat 23 might lead to conformational changes within FLNa that reduce its binding affinity to the cytoplasmatic domain of the β2-chain of LFA-1.

The current models for LFA-1 affinity modulation suggest that inside-out signals mediate direct interaction of Talin and Kindlin-3 to the cytoplasmic domain of CD18 separating the α/β cytoplasmic tails. The binding of these molecules induces changes within the integrin structure resulting in a shift from the low affinity to intermediate and high affinity conformation (1, 2). Talin was shown to induce the intermediate conformation (9) whereas Kindlin-3 was required for the high affinity conformation and stabilization of LFA-1/ICAM-1 binding (66). SKAP55 was described to interact with RIAM that binds to Talin (18, 24) or with RAPL linked to Mst1 (25, 31). RAPL was shown to bind to the cytoplasmic domain of CD11a (16). It might be that activated Ndr2 (through Mst1 and Mob1) alone or in the context with SKAP55/RAPL/Mst1/Mob1-complex bound to the cytoplasmic domain of CD11a is a prerequisite for FLNa phosphorylation at S2152 and its release. Subsequently, SKAP55 bound via RIAM to Talin interacting with CD18 induces the intermediate conformation, which in turn allows Kindlin-3 (either sequentially or simultaneously) binding to CD18 stabilizing the high affinity conformation of LFA-1.

The high affinity conformation of LFA-1 mediates T-cell adhesion (and interaction with APCs). Similar to affinity regulation avidity modulation (clustering) has been thought to increase contact numbers and stabilize adhesion. However, recently Raab et al. showed that clustering and cross-linking of LFA-1 during maturation of the IS reduces or terminates adhesion (or interaction with APCs) (29). They proposed a model that cross-linked LFA-1 activates Focal Adhesion Kinase 1 (FAK1), which phosphorylates the linker for the activation of T cells (LAT) at Y171. This pY171 provides a docking site for a complex consisting of growth-factor-receptor bound protein 2 (Grb2) and SKAP55 (29). As depicted in Figures 2 and 5 our data reveal that Ndr2 provides a positive regulatory signal for affinity modulation of LFA-1 leading to adhesion and activation of T cells. Since Mst1-deficient T cells exhibit a defect in LFA-1 clustering (32) it is likely that activated Ndr2 through Mst1/Mob1 modulates LFA-1 clustering and FAK1 activity to terminate adhesion.

Previous studies using Talin-deficient CD4^+^ T cells or CD4^+^ T cells expressing a LFA-1 mutant deficient in Kindlin-3 binding showed an attenuated antigen-specific CD4^+^ T-cell activation *in vivo* (9, 67). Therefore it is likely that loss of Ndr2 in CD4^+^ T cells would also interfere with interaction of APCs to promote T-cell activation and proliferation *in vivo*. Clearly further experiments are required to address these issues.

Based on these observations we suggest the following molecular interaction model (Figure S7): TCR-mediated activation of Ndr2 probably by Mst1/2 kinases (step 1) leads to phosphorylation of FLNa at S2152 (step 2). This phosphorylation event releases FLNa from LFA-1 (step 3), thereby allowing the association of Talin and Kindlin-3 with LFA-1 (step 4). The inducible interaction with Talin and Kindlin-3 will mediate the induction of the high affinity status of LFA-1 (step 5). Strikingly, the activation dynamics of Ndr2 are comparable to the increase of FLNa phosphorylation at S2152 and Talin/Kindlin-3 association with LFA-1, all of which can be detected within 5 min after TCR stimulation. However, Ndr2 activity appears to decay faster than the FLNa phosphorylation levels of S2152 and the formation of the LFA-1/Talin/Kindlin complex indicating that Ndr2 is critical for the initiation of these processes but additional factors are involved in the maintenance of LFA-1 open conformation and FLNa phosphorylation.

In addition to Ndr2, the p21-activated kinase 1 (Pak1) has been described to phosphorylate FLNa at S2152 *in vitro* and *in vivo* (68). This kinase is recruited to the immunological synapse (IS) (69, 70). In Jurkat T cells a trimolecular complex was identified consisting of Pak, the guanine nucleotide exchange factor PIX and the ADF-ribosylation factor-activating protein (Arf-GAP) GIT (69). The recruitment of Pak to the IS is dependent on the interaction with PIX and is also important for its activation (69). Evidence that activated Pak might be involved in TCR-mediated LFA-1 activation is shown by the study of Missi (70). Here the authors analyzed α-PIX-deficient T cells. Molecular events that were altered in these lymphocytes include decreased GIT2 (an isoform of GIT) expression, Pak phosphorylation and recruitment of Pak and LFA-1 to the IS (70). The reduced LFA-1 in the IS correlates with an attenuated interaction of α-PIX-deficient T cells with APCs (70). Pak1 might also indirectly regulate TCR-mediated LFA-1 activation and adhesion through activation of phospholipase C gamma 1 (PLCγ1) to promote Rap1 activation. The disruption of the PIX-Pak interaction or the overexpression of a dominant negative mutant of Pak attenuated PLCγ1 phosphorylation upon stimulation with superantigen-loaded B cells (69) or after TCR triggering with antibodies (71). PLCγ1 hydrolyzes phosphatidylinositol 4,5-biphosphate to produce diacylglycerol (DAG) and Inositol triphosphate (IP_3_), which in turn increases the level of intracellular free calcium (72). Katagiri and colleagues identified a guanine nucleotide exchange factor that contains both calcium and DAG binding domains (CalDAG-GEFI) downstream of PLCγ1 that regulated TCR-mediated Rap1 activation to facilitate T-cell adhesion (73). However, whether Pak1 regulates TCR-mediated adhesion independently of FLNa phosphorylation at S2152 via Rap1 activation or is interrelated with the Ndr2-pathway of FLNa phosphorylation clearly requires further investigations.

In conclusion we demonstrate that the serine/threonine kinase Ndr2, in addition to its critical role for apoptosis, cell polarity and proliferation (34, 35), is indispensable for TCR-induced LFA-1 activation. Ndr2-dependent phosphorylation of FLNa at S2152 seems to be the initial step of LFA-1 activation to switch from the closed to the open high affinity conformation of this integrin required for ligand binding.

## Author Contributions

BS, OS, and SK conceived and designed the research. NW, AS, CM, AR, and YD designed and performed experiments, and analyzed data. ED and BT performed and characterized Ndr phosphorylation specificity. CF provided purified GST, GST-CD18_cyt_, and GST Igl repeats 19–24 of human FLNa fusion proteins. PR and AM prepared T/B cell pairs for confocal microscopy. BS, OS, and SK prepared the manuscript.

### Conflict of Interest Statement

The authors declare that the research was conducted in the absence of any commercial or financial relationships that could be construed as a potential conflict of interest.
