# Supplementary material for: Filamin A Phosphorylation at Serine 2152 by the Serine/Threonine Kinase Ndr2 Controls TCR-Induced LFA-1 Activation in T Cells
Source: Front Immunol. 2018 Dec 4;9:2852. doi: 10.3389/fimmu.2018.02852 (PMC6290345; doi:10.3389/fimmu.2018.02852)
Supplement: Supplementary file 1 [file Data_Sheet_1.pdf]

Supplemental Information

Filamin A phosphorylation at serine 2152 by the serine/threonine kinase Ndr2 controls TCR-induced LFA-1 activation in T cells

Natalie Waldt, Anke Seifert, Yunus Emre Demiray, Eric Devroe, Benjamin E. Turk, Peter Reichardt, Charlie Mix, Annegret Reinhold, Christian Freund, Andreas J. Müller, Burkhart Schraven, Oliver Stork, and Stefanie Kliche

Supplemental Information

Table S1

Name of the primer	Sequence of the primer
Renilla shRNA for	5'-GATCCCC**CCAAGTAATGTAGGATCAA**TTCAAGAGATTGATCCTACATTACTTGGTTTTTA-3' (Horn et al., 2009)
Renilla shRNA rev	5'-AGCTTAAAAA CCAAGTAATGTAGGATCAA TCTCTTGAA TTGATCCTACATTACTTGGGGG-3'
FLNa shRNA for	5'-GATCCCC**CCCACCCACTTCACAGTAAAT**TTCAAGAGAATTTACTGTGAAGTGGGTGG GTTTTTA-3' (Yuan et al., 2004)
FLNa shRNA rev	5'-AGCTTAAAAACCCACCCACTTCACAGTAAATTCTCTTGAAATTTACTGTGAAGTGGGTGG GGGG-3'
Ndr2-shRNA for	5'-GATCCCC**GAGGAAACACAGTTCTACA**TTCAAGAGATGTAGAACTGTGTTTCCTCTTTTTGG AAA-3'
Ndr2 shRNA rev	5'-AGCTTTTCCAAAAA GAGGAAACACAGTTCTACA TCTCTTGAA TGTAGAACTGTGTTTCCTC GGG-3'
Ndr2 sh-res for	5'-GACACCTTGACAGAA GAaGAgACgCAaTTtTAtA TTTCAGAGACTGTTCTGGCAATAGATGCG-3'
Ndr2 sh-res rev	5'-CGCATCTATTGCCAGAACAGTCTCTGAAATATAAAATTGCGTCTCTTCTTCTGTCAAGG TGTC-3'
Ndr2 K119A for	5'-ACAGGCCATATCTATGCAATG gcG ATATTGAGAAAGTCTGATATG-3'
Ndr2 K119A rev	5'-CATATCAGACTTTCTCAATATCGCACTTGCATAGATATGGCCTGT-3'
Ndr2 BglII for	5'-CCCAGATCTATGGCAATGACGGCAGGGACT-3'#
Ndr2 SalI/Xba1 rev	5'-CCCGTCGACTCTAGATCATAACTTCCCAGCTTT-3'#
Ndr2 MluI for	5'-GCGCGCACGCGTGCCACCATGGCAATGACGGCAGGGACT-3'#
Ndr2 NotI for	5'-GCGCGCGCGGCCGCTTCATAACTTCCCAGCTT-5'#
Ndr1 BglII for	5'-CCCAGATCTATGGCAATGACAGGCTCAACACCT-3'#
Ndr1 SalI/Xba1 rev	5'-CCCGTCGACTCTAGACTATTTTGCTGCTTTCAT-3'#

in bold: shRNA target sequence

* Lowercase letters indicate changed nucleotides

Underlined sequences indicate restriction sites

Figure S1

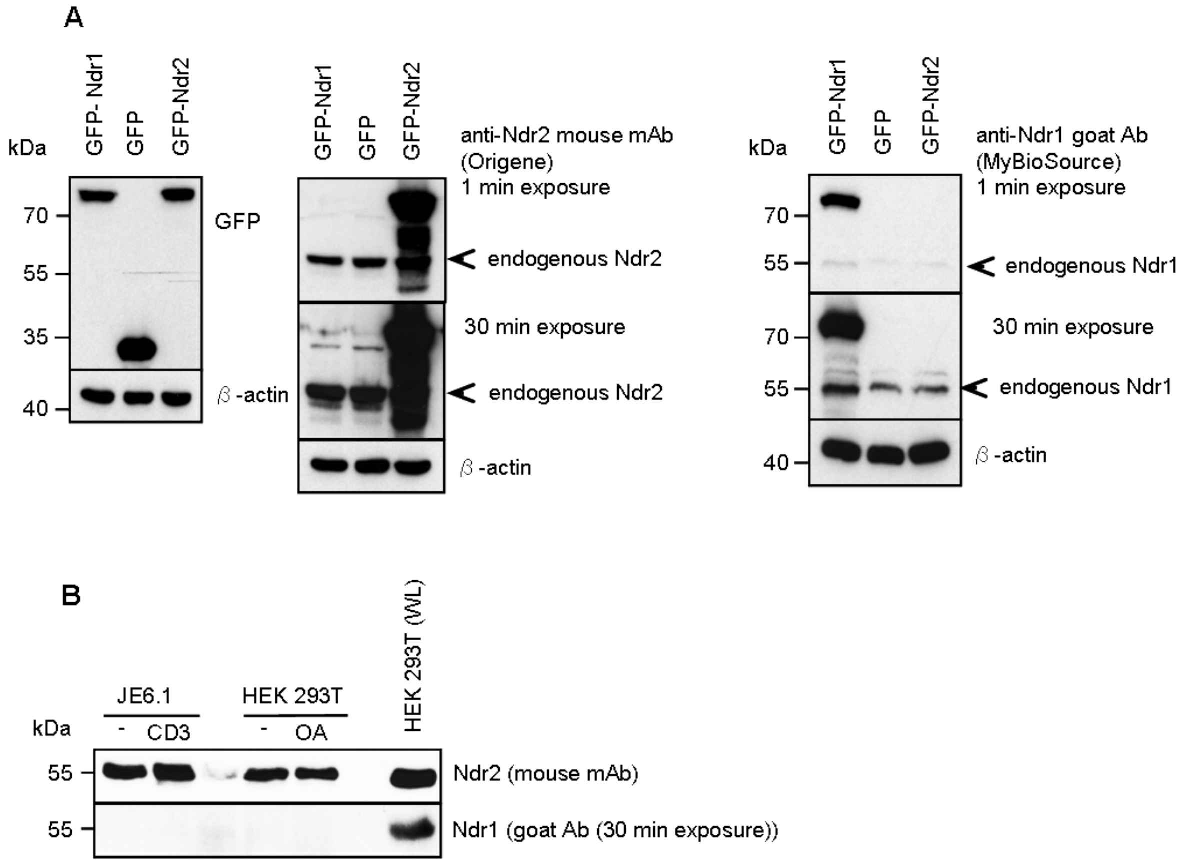
A
kDa
GFP- Ndr1
GFP
GFP-Ndr2
70
55
35
40
GFP
β -actin
GFP-Ndr1
GFP
GFP-Ndr2
anti-Ndr2 mouse mAb
(Origene)
1 min exposure
endogenous Ndr2
30 min exposure
endogenous Ndr2
β -actin
kDa
GFP-Ndr1
GFP
GFP-Ndr2
anti-Ndr1 goat Ab
(MyBioSource)
1 min exposure
70
55
endogenous Ndr1
70
30 min exposure
55
endogenous Ndr1
40
β -actin
B
HEK 293T (WL)
JE6.1
HEK 293T
kDa
-
CD3
-
OA
55
Ndr2 (mouse mAb)
55
Ndr1 (goat Ab (30 min exposure))

Figure S2

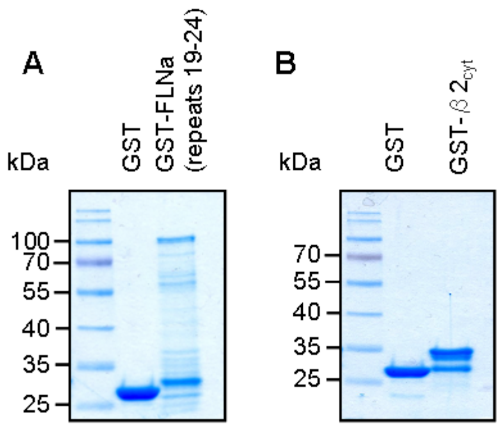
A
kDa
GST
GST-FLNa
(repeats 19-24)
100
70
55
40
35
25
B
kDa
GST
GST-β 2cyt
70
55
40
35
25

Figure S3

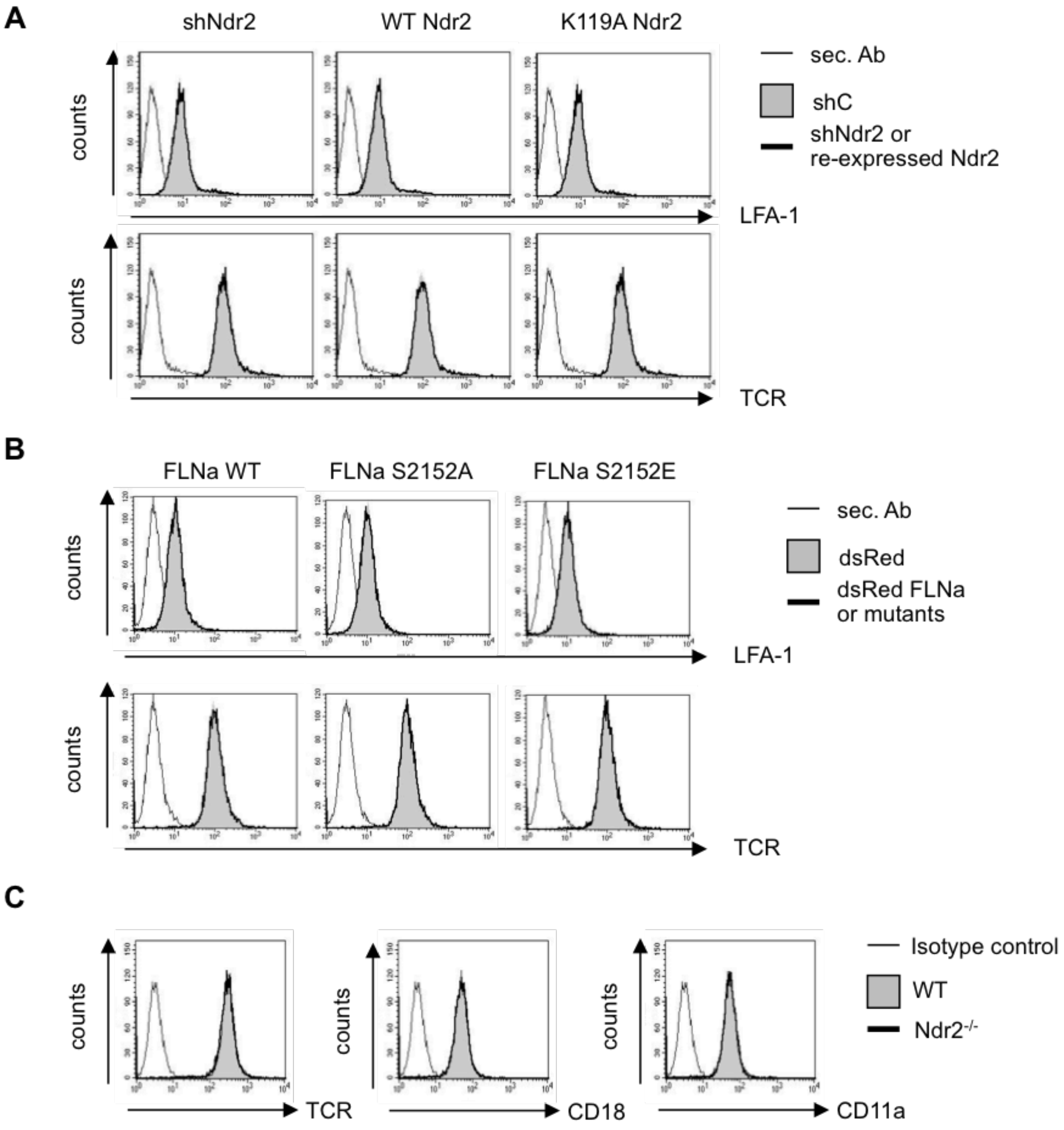
A
shNdr2
WT Ndr2
K119A Ndr2
counts
sec. Ab
shC
shNdr2 or
re-expressed Ndr2
LFA-1
TCR
B
FLNa WT
FLNa S2152A
FLNa S2152E
sec. Ab
dsRed
dsRed FLNa
or mutants
C
Isotype control
WT
Ndr2-/-
CD18
CD11a

Figure S4

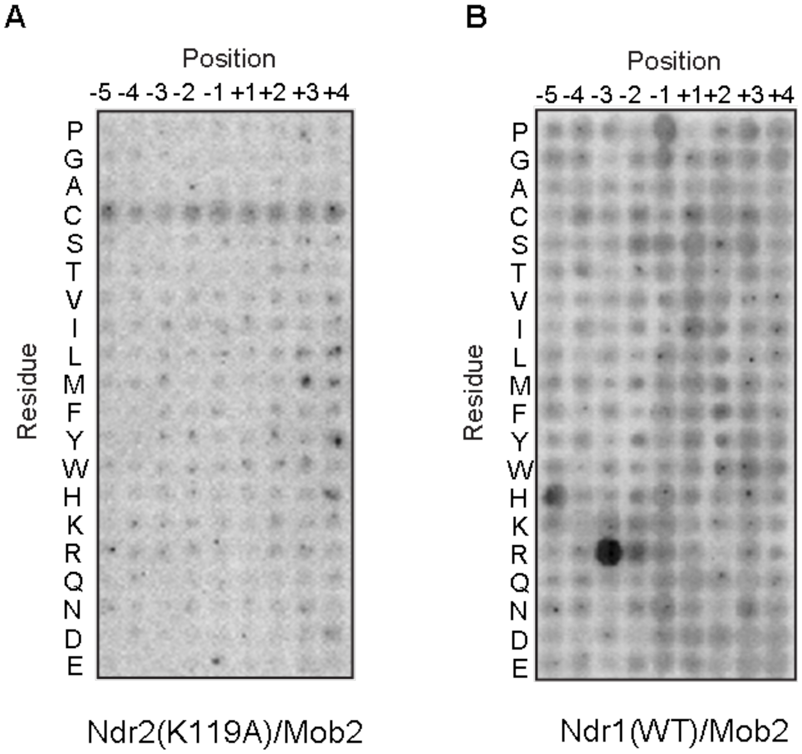
A
Position
-5 -4 -3 -2 -1 +1 +2 +3 +4
Residue
P G A C S T V I L M F Y W H K R Q N D E
Ndr2(K119A)/Mob2
B
Position
-5 -4 -3 -2 -1 +1 +2 +3 +4
Residue
P G A C S T V I L M F Y W H K R Q N D E
Ndr1(WT)/Mob2

Figure S5

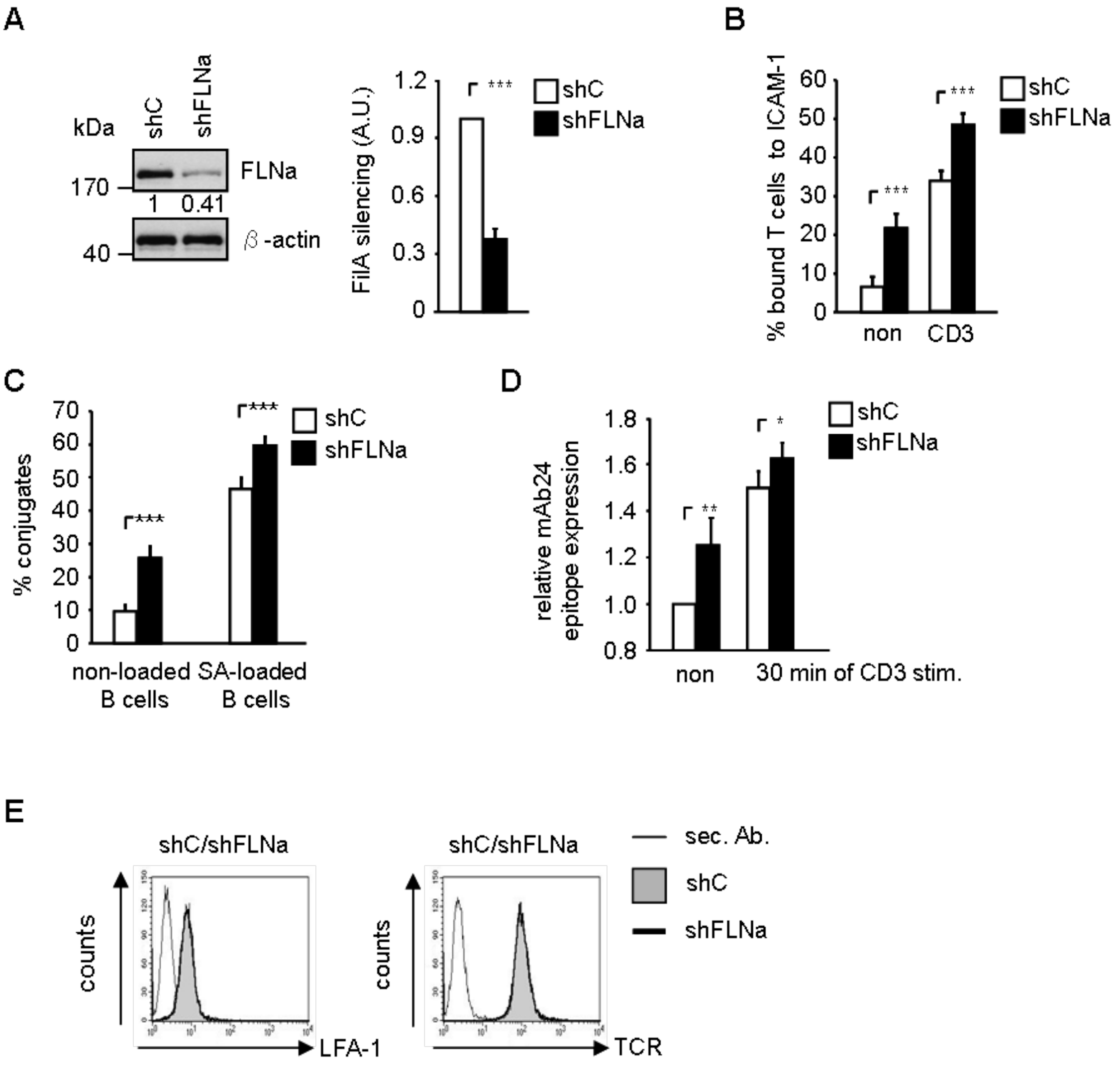
A
kDa
shC
shFLNa
170
40
FLNa
1 0.41
β-actin
FilA silencing (A.U.)
1.2
0.9
0.6
0.3
0
***
shC
shFLNa
B
% bound T cells to ICAM-1
60
50
40
30
20
10
0
***
***
non
CD3
shC
shFLNa
C
% conjugates
70
60
50
40
30
20
10
0
***
***
non-loaded B cells
SA-loaded B cells
shC
shFLNa
D
relative mAb24 epitope expression
1.8
1.6
1.4
1.2
1.0
0.8
**
*
non
30 min of CD3 stim.
shC
shFLNa
E
shC/shFLNa
shC/shFLNa
counts
counts
LFA-1
TCR
sec. Ab.
shC
shFLNa

Figure S6

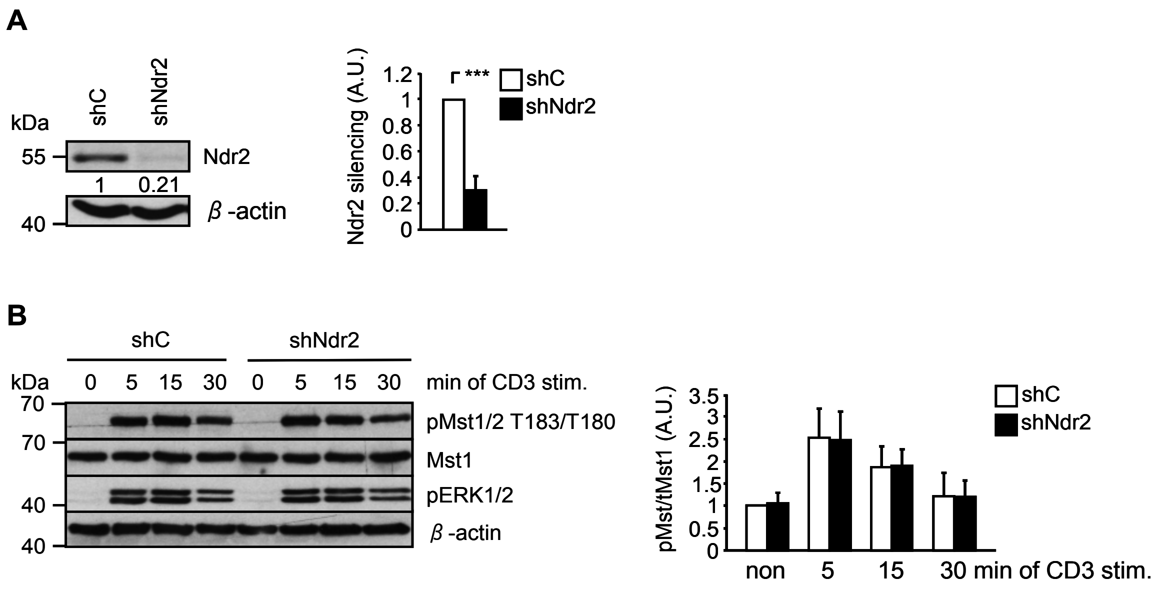
A
kDa
shC
shNdr2
55
40
Ndr2
1
0.21
β-actin
Ndr2 silencing (A.U.)
1.2
1
0.8
0.6
0.4
0.2
0
***
shC
shNdr2
B
shC
shNdr2
kDa
0
5
15
30
0
5
15
30
min of CD3 stim.
70
70
40
40
pMst1/2 T183/T180
Mst1
pERK1/2
β-actin
pMst/tMst1 (A.U.)
3.5
3
2.5
2
1.5
1
0.5
0
non
5
15
30 min of CD3 stim.
shC
shNdr2

Figure S7

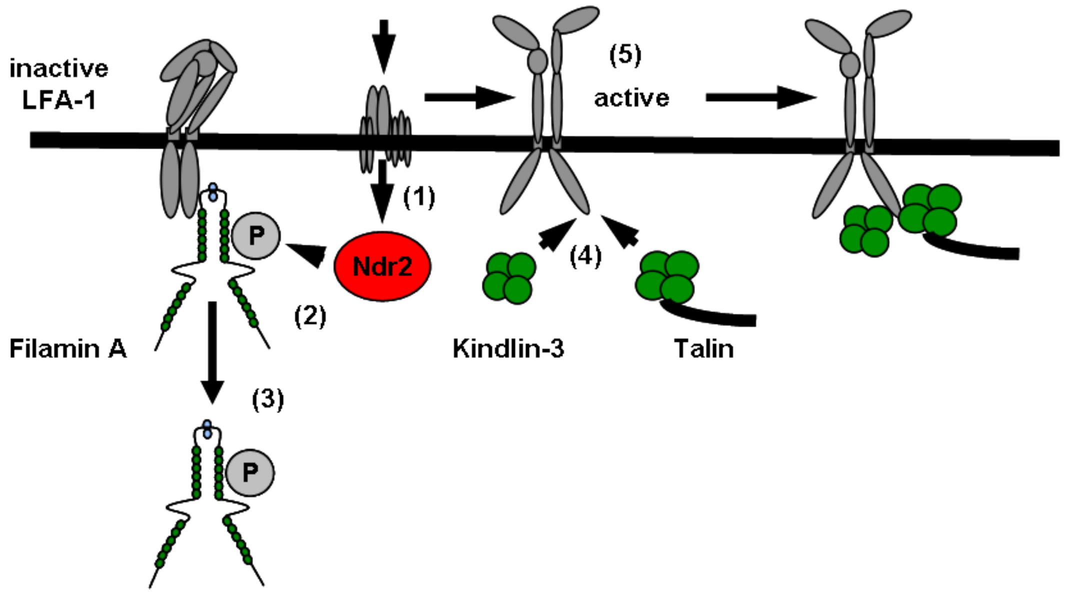
inactive
LFA-1
(1)
Ndr2
P
(2)
Filamin A
(3)
P
(5)
active
(4)
Kindlin-3
Talin

Figure S8

A

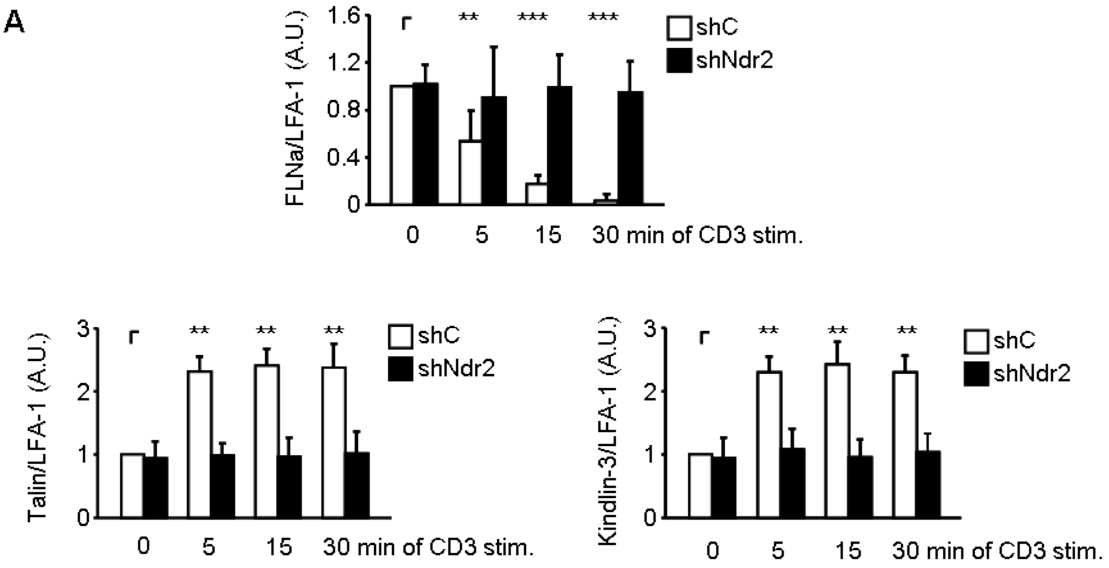
FLNa/LFA-1 (A.U.)
1.6
1.2
0.8
0.4
0
** *** ***
shC
shNdr2
0 5 15 30 min of CD3 stim.
Talin/LFA-1 (A.U.)
3
2
1
0
** ** **
shC
shNdr2
0 5 15 30 min of CD3 stim.
Kindlin-3/LFA-1 (A.U.)
3
2
1
0
** ** **
shC
shNdr2
0 5 15 30 min of CD3 stim.

B

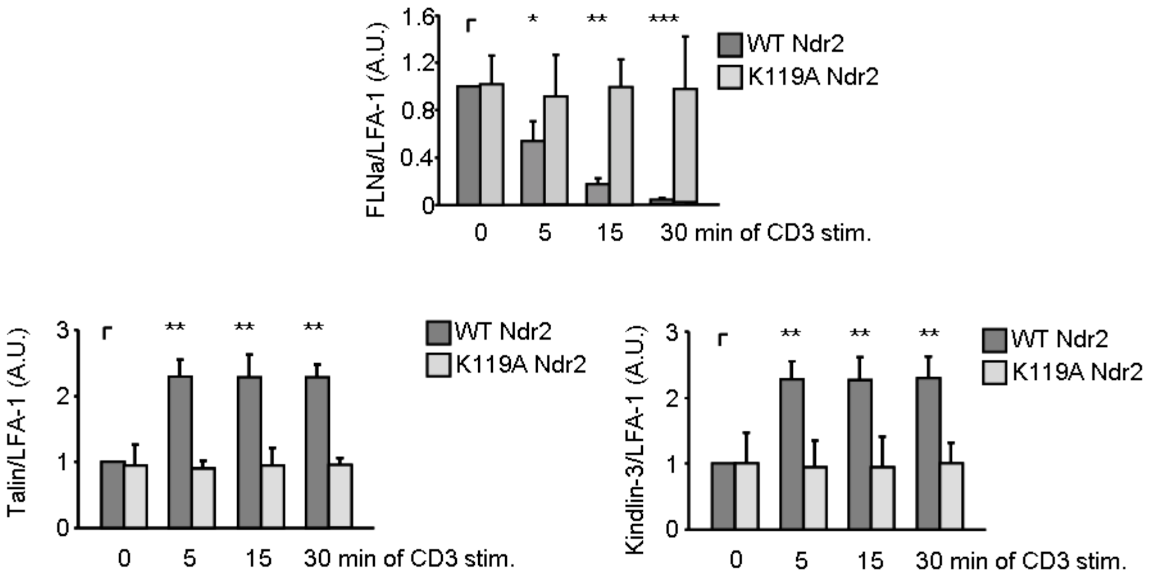
FLNa/LFA-1 (A.U.)
1.6
1.2
0.8
0.4
0
* ** ***
WT Ndr2
K119A Ndr2
0 5 15 30 min of CD3 stim.
Talin/LFA-1 (A.U.)
3
2
1
0
** ** **
WT Ndr2
K119A Ndr2
0 5 15 30 min of CD3 stim.
Kindlin-3/LFA-1 (A.U.)
3
2
1
0
** ** **
WT Ndr2
K119A Ndr2
0 5 15 30 min of CD3 stim.

Supplemental Information

Figure S1. Specificity of Ndr2 and Ndr1 antibodies used in this study

(A) HEK 293T cells were transfected with plasmids encoding for GFP, wild type GFP-tagged Ndr1 (GFP-Ndr1) or GFP-tagged WT Ndr2 (GFP-Ndr2). Lysates from these transfectants were prepared and used to address the specificity of antibodies to detect GFP, Ndr1 or Ndr2 by Western blotting. Arrows indicate the position of endogenous Ndr1 or Ndr2 expressed in this cell line (n=2). (B) The anti-Ndr2 rabbit Ab was used to immunoprecipitate endogenous Ndr2 from lysates of unstimulated or anti-CD3-stimulated (CD3) Jurkat T cells and non-treated or okadaic acid (OA)-treated HEK 293T cells. Precipitates were analyzed by Western blotting with the indicated antibodies (n=2). The data shown here indicate that all antibodies used in this study were specific to detect either Ndr2 or Ndr1, respectively.

Figure S2. Purity of GST-fusion proteins used in this study

(A) 5µg of GST and GST-fusion protein of a C-terminal fragment of human FLNa (repeats 19–24; GST-FilA) were analyzed by SDS-PAGE and proteins were visualized by Coomassie blue staining (n=3). (B) 10 µg of GST or GST-fusion protein of the cytoplasmic domain of CD18 (GST-$CD18_{cyt}$) were separated on a SDS-PAGE and proteins were visualized by Coomassie blue staining (n=3).

Figure S3. Expression levels of the TCR, CD18 and CD11a of transfected Jurkat T cells or mouse $CD4^+$ T cells (A) Jurkat T cells were transfected with suppression/re-expression constructs that do not suppress endogenous Ndr2 (shC), or reduces the protein level of Ndr2 (shNdr2) and re-express a FLAG-tagged shRNA-resistant wild type form of Ndr2 (WT Ndr2) or the kinase dead mutant (K119A Ndr2). Transfectants were used for flow cytometric analysis of the TCR and CD18 surface expression within the GFP gate (n=4). (B) Jurkat T cells were transiently transfected with dsRed C1 (vector) or plasmids encoding wild type (WT) dsRed-tagged FLNa (FLNa WT) or dsRed-tagged S2152A or dsRed-tagged S2152E FLNa mutants (FLNa S215A and FLNa S2152E). After 24h transfectants were used for flow cytometric analysis of the TCR and CD18 surface expression within the dsRed gate (n=4). (C) Purified splenic WT or $Ndr2^{-/-}$ $CD4^+$ T cells were used for flow cytometric analysis of the TCR, CD18 and CD11a within the $CD4^+$ positive gate. One representative experiment out of three is shown.

Figure S4. Peptide array analysis of Ndr1 and kinase inactive Ndr2

(A) Kinase inactive Ndr2(K119A)-Mob2 complex and (B) WT Ndr1-Mob2 complex were used as in **Figure 4** to phosphorylate the arrayed positional scanning peptide library (n=2).

Figure S5. Silencing of FLNa enhances TCR-mediated LFA-1 activation

(A) Jurkat T cells were transfected with suppression plasmids which do not suppress endogenous FLNa (shC), or reduce the endogenous protein level of FLNa (shFLNa). 48 hour after transfection, lysates were analyzed by Western blotting for FLNa and β-actin expression. Quantification of knockdown efficiency is presented in arbitrary units (A. U.) Intensity of FLNa-bands of all samples were normalized to FLNa samples from shC-transfected cells which were set to 1 (n=4). (B) Transfectants were left untreated (non) or stimulated for 30 min with anti-CD3 antibodies (CD3). Cells were analyzed for adhesion to ICAM-1-coated 96 well plates. Bound cells were counted and calculated as percentage of input (n=4). (C) Cells were transfected as described in (A) and analyzed for their ability to form conjugates with DDAO-SE (red)-stained Raji B cells that were pulsed without (non) or with superantigen (SA) for 30min at 37°C. The percentage of conjugates was defined as the number of double-positive events in the upper right quadrant (n=4). (D) Jurkat T cells transfected as described in (A) were left untreated (non) or stimulated with anti-CD3 antibodies (CD3), followed by staining with the anti-LFA-1 antibody mAb24 which recognizes the high affinity conformation of LFA-1. mAb24 epitope expression was assessed by flow cytometry and data are normalized against LFA-1 expression detected by MEM48 (n=4). (E) Transfectants as described in (A) were used for flow cytometric analysis of the TCR and CD18 surface expression within the GFP gate (n=3). (mean± SEM *$p \leq 0.05$; **$p \leq 0.01$ ***$p \leq 0.001$)

Figure S6. Suppression of Ndr2 does not abrogate TCR-mediated Mst1/2 activation.

(A) Jurkat T cells were transfected with suppression plasmids which do not suppress endogenous Ndr2 (shC), or reduce the endogenous protein level of Ndr2 (shNdr2). 48 hour after transfection, lysates were analyzed by Western blotting for Ndr2 and β-actin expression. Quantification of knockdown efficiency is presented in arbitrary units (A. U.). Intensity of Ndr2-bands of all samples was normalized to Ndr2 samples from shC-transfected cells which were set to 1 (n=4; lower graph). (B) Cells transfected as described in A were left untreated or were stimulated with CD3 antibodies for the indicated periods of time. Lysates were analyzed for the activation status of Mst1/2 and the expression levels of Mst1 by Western blotting using the indicated antibodies (n=4). Densitrometric analysis of phosphorylated Mst1/2 (pMst) normalized to total Mst1 (tMst1) (n=4; right graph).

Figure S7. Model of Ndr2-controlled LFA-1 activation

TCR-induced activation of Ndr2 (step 1) promotes FLNa phosphorylation at S2152 (step 2) resulting in release of FLNa from LFA-1 (step 3). This in turn allows the association of Talin and Kindlin-3 with LFA-1 (step 4) thereby inducing full LFA-1 activation (step 5).

Figure S8. Densitrometric analysis of FLNa, Talin or Kindlin-3 association to LFA-1

Densitrometric analysis of FLNa, Talin or Kindlin-3 bound to LFA-1 as depicted in **Figures 7** B and 7C (n=3). (mean ± SEM; *p≤0.05, **p≤0.01, ***p≤0.001)

References

Horn J, Wang X, Reichardt P, Stradal TE, Warnecke N, Simeoni L, et al. Src homology 2-domain containing leukocyte-specific phosphoprotein of 76 kDa is mandatory for TCR-mediated inside-out signaling, but dispensable for CXCR4-mediated LFA-1 activation, adhesion, and migration of T cells. *J Immunol* (2009) 183:5756-67. doi:10.4049/jimmunol.0900649.

Yuan B, Latek R, Hossbach M, Tuschl T, Lewitter F. siRNA Selection Server: an automated siRNA oligonucleotide prediction server. *Nucleic Acids Res* (2004) 32 Server issue) W130-134.
